# A standardised protocol for relative SARS-CoV-2 variant severity assessment, applied to Omicron BA.1 and Delta in six European countries, October 2021 to February 2022

**DOI:** 10.2807/1560-7917.ES.2023.28.36.2300048

**Published:** 2023-09-07

**Authors:** Tommy Nyberg, Peter Bager, Ingrid Bech Svalgaard, Dritan Bejko, Nick Bundle, Josie Evans, Tyra Grove Krause, Jim McMenamin, Joël Mossong, Heather Mutch, Ajibola Omokanye, André Peralta-Santos, Pedro Pinto-Leite, Jostein Starrfelt, Simon Thelwall, Lamprini Veneti, Robert Whittaker, John Wood, Richard Pebody, Anne M Presanis

**Affiliations:** 1 MRC Biostatistics Unit, University of Cambridge, Cambridge, United Kingdom; 2 Statens Serum Institut, Copenhagen, Denmark; 3 Health Directorate, Luxembourg, Luxembourg; 4 European Centre for Disease Prevention and Control, Stockholm, Sweden; 5 Public Health Scotland, Glasgow, Scotland, United Kingdom; 6 Directorate-General of Health, Lisbon, Portugal; 7 Norwegian Institute of Public Health, Oslo, Norway; 8 COVID-19 Vaccines and Epidemiology Division, UK Health Security Agency, London, United Kingdom; 9 World Health Organization Regional Office for Europe, Copenhagen, Denmark; * These authors contributed equally to this work and share last authorship.

**Keywords:** SARS-CoV-2, COVID-19, severity, virulence, variant of concern, study protocol

## Abstract

Several SARS-CoV-2 variants that evolved during the COVID-19 pandemic have appeared to differ in severity, based on analyses of single-country datasets. With decreased testing and sequencing, international collaborative studies will become increasingly important for timely assessment of the severity of new variants. Therefore, a joint WHO Regional Office for Europe and ECDC working group was formed to produce and pilot a standardised study protocol to estimate relative case-severity of SARS-CoV-2 variants during periods when two variants were co-circulating. The study protocol and its associated statistical analysis code was applied by investigators in Denmark, England, Luxembourg, Norway, Portugal and Scotland to assess the severity of cases with the Omicron BA.1 virus variant relative to Delta. After pooling estimates using meta-analysis methods (random effects estimates), the risk of hospital admission (adjusted hazard ratio (aHR) = 0.41; 95% confidence interval (CI): 0.31−0.54), admission to intensive care unit (aHR = 0.12; 95% CI: 0.05−0.27) and death (aHR = 0.31; 95% CI: 0.28−0.35) was lower for Omicron BA.1 compared with Delta cases. The aHRs varied by age group and vaccination status. In conclusion, this study demonstrates the feasibility of conducting variant severity analyses in a multinational collaborative framework and adds evidence for the reduced severity of the Omicron BA.1 variant.

## Introduction

During the COVID-19 pandemic, several variants of the severe acute respiratory syndrome coronavirus 2 (SARS-CoV-2) evolved. These had different risks of causing severe disease [1-18], possibly due to differences in symptom profile [19-22], viral load [23,24], vaccine effectiveness [3,5,22,25,26] or via other mechanisms. A wide range of estimates of the relative risks of severe outcomes such as hospitalisation and death associated with new variants have been reported, often with moderate precision. Some countries have published variant severity assessments based on national data, but many countries have not. Thus, there are likely observational data available on COVID-19 cases and their outcomes which could further inform about variant severity, if analysed within a valid study design and statistical analysis framework to minimise potential biases.

During the spring and summer of 2022, mass testing for COVID-19, as well as sequencing capacity, was reduced in many countries where the pandemic appeared to recede [27-30]. As data on cases and the virus variants become less available, collaborative international efforts will become more important. Such collaborations can provide larger effective sample sizes than those available in single-country datasets, which may enable more rapid identification of differences in virulence between virus variants.

To address these issues, a Joint World Health Organization (WHO) Regional Office for Europe and European Centre for Disease Prevention and Control (ECDC) Infection Severity Working Group was formed. This was an ad hoc group comprised of public health agencies and academic collaborators in countries working on assessing differences in severity of COVID-19 by virus variants. The aim of the group was to produce a standardised protocol to be used by investigators in individual countries to analyse locally available data on COVID-19 cases using comparable methods and definitions. This decentralised approach overcomes potential issues in sharing individual-level data between countries. The intended application of the standardised protocol is for the comparison of the disease risks between two SARS-CoV-2 variants, during calendar periods when both variants are circulating and individual-level data on which virus variant caused the infection of each case are available. Outcomes include indicators of severe disease, such as hospital admission, intensive care unit (ICU) admission or death. The objective was to assess differences in the severity of COVID-19 by virus variants, but the approach is applicable also to the study of severity of other similar diseases, such as influenza, by various virus subtypes. This approach enables direct contrasting of relative risk (RR) estimates based on consistently analysed data from several countries and allows international organisations and researchers to pool those estimates. Pooled estimates can provide greater precision due to a larger sample size compared with estimates from separate countries.

The primary aim of this report was to describe the development of the standardised protocol. The protocol was further applied in a pilot study of data on the full national cohorts of cases with Delta (Phylogenetic Assignment of Named Global Outbreak (Pango) lineage designation B.1.617.2) and Omicron (Pango lineage designation B.1.1.529) BA.1 variants from six European countries.

## Protocol development and revision

**Figure 1:**
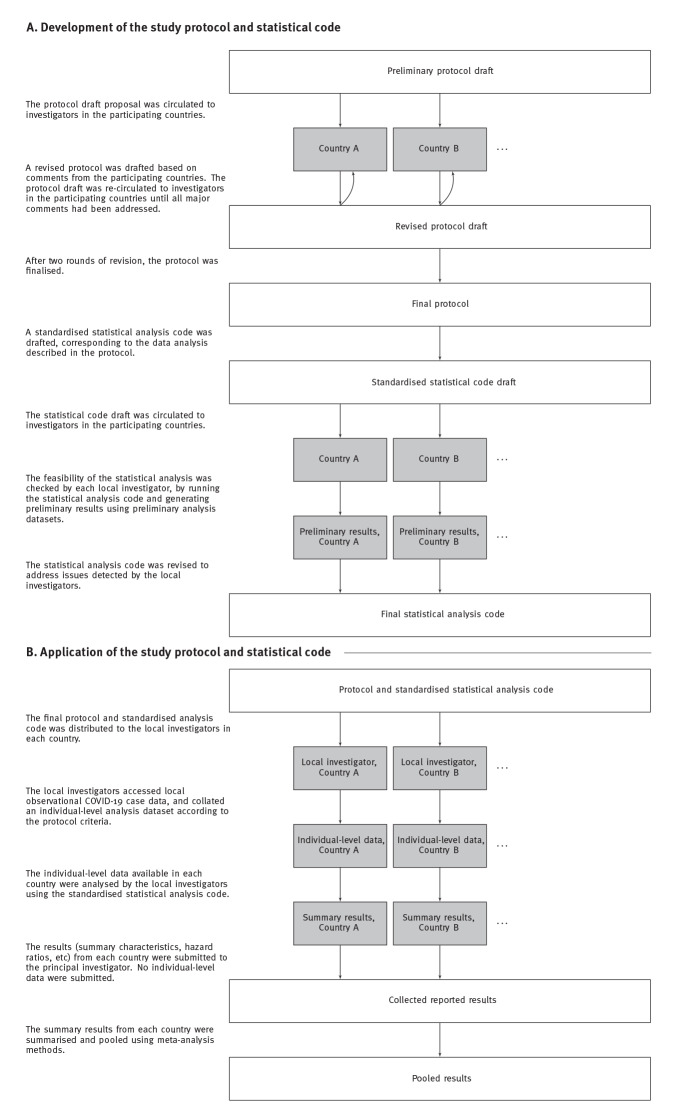
Summary of the development and application of the study protocol and the statistical analysis code

The development of the study protocol and the associated statistical analysis code are summarised in Figure 1A. A first version of the protocol was drafted and circulated for comments from the six countries participating in the working group (national public health agencies in Denmark, England, Luxembourg, Norway, Portugal and Scotland), together with a survey on the testing, variant characterisation and outcome data available in each country. This survey and ensuing discussions identified a need to simplify the original inclusion criteria. Initially, the inclusion period was based on a required minimum observed prevalence of each variant, but this was changed to a recommendation to choose a consecutive period when cases of each variant were prevalent, at the discretion of the local investigators. For the potential confounders, the revised protocol acknowledged that vaccination status should be defined to reflect the categories (number of vaccine doses and time since last dose) applicable in the study inclusion period. These changes resulted in a second revised protocol. After the second protocol had been circulated and approved, the country-level statistical analysis described in the protocol was translated into a standardised statistical analysis code, available at: https://github.com/TommyNyberg/variant_severity. The approved study protocol is available in Supplement 1.

The study protocol and standardised statistical analysis code were created and applied in a pilot study to estimate differences in COVID-19 severity outcomes for cases infected with the Omicron BA.1 compared with the Delta variant in six European countries. The full protocol is available in Supplement 1.

## Standardised data analysis including hazard ratio estimation

In the pilot study, the feasibility of performing the standardised analysis according to the study protocol was assessed. For this, each country used the statistical analysis code to analyse their national data on identified cases infected with the Delta or Omicron BA.1 variants.

The full protocol is available in the Supplementary material (Supplement 1). In brief, retrospective cohorts of COVID-19 cases infected with Delta or Omicron BA.1 were identified by the local investigators. For inclusion, data on the SARS-CoV-2 variant of each case were required, for example through whole genome sequencing of swab samples taken for PCR testing or using other methods such as reverse transcription-quantitative PCR (RT-qPCR) [31] or S gene target surveillance [32]. The protocol recommended the inclusion period be restricted to a period when the observed cases included both cases with Delta infections and cases with Omicron BA.1 infections. If a characterisation method with lower sensitivity or specificity than sequencing such as S gene target surveillance was used, the protocol further recommended restricting the inclusion period to the earliest and latest date when variant prevalence was sufficiently high to ensure predictive values > 90% for both variants. Otherwise, the choice of the inclusion period was left to the local investigators.

Hazard ratios (HRs) of severe outcomes following detection of Omicron BA.1 or Delta were estimated with Cox proportional hazards regression models, stratified by vaccination status (unvaccinated, ≥ 0 days after first dose, ≥ 14 days after second dose, ≥ 153 days after second dose, ≥ 14 days after third dose), region (allowed to differ according to national circumstances) and time period of positive test sample, and adjusted for age (age bands 0–19, 20–39, 40–49, 50–59, 60–69 and ≥ 70 years, with additional regression adjustment for within-age-band exact age by the inclusion of separate age-band-specific linear effect terms for quantitative age) and sex (binary variable) at a minimum (‘required adjustment variables’). Two adjustments were considered for the time periods: one where the stratification was for an exact date and another where the stratification was for a calendar week with additional regression adjustment for an exact date within each week. If available, adjustments for reinfection status (previous positive test ≥ 90 days before current episode, ‘highly desired adjustment variable’) and ethnicity, socioeconomic status, comorbidity and international travel (all allowed to differ according to which data were available nationally, ‘desired adjustment variables’) were carried out in further analyses. Each of the three severe outcomes, if available, were considered: hospital admission within 14 days of a positive test, ICU admission within 14 days of a positive test and death within 28 days of a positive test. When available, the investigators were requested to provide results for hospital admissions, ICU admissions or deaths both due to COVID-19 (COVID-19-specific outcomes) and among people with COVID-19 (all-cause outcomes), according to national criteria. Subgroup analyses by age group and vaccination status were also requested.

As a sensitivity analysis, the protocol further requested assessing the potential impact of epidemic phase bias, a bias that may occur when comparing two virus variants in different phases of growth or decline [33].

## Meta-analysis of hazard ratio estimates

Using meta-analysis methods, the HR estimates from each country were contrasted and the heterogeneity between estimates was assessed using the *I* ^2^ statistic (proportion of the pooled hazard ratio variance that is attributable to between-country heterogeneity). If countries had reported HRs of COVID-19-specific outcomes, these were used in the meta-analysis, and otherwise HRs of all-cause outcomes. The HRs were pooled using both fixed effects and random effects models. A supplementary power calculation was performed to explore the statistical power of a multinational meta-analysis by number of countries, sample size and between-country heterogeneity.

The country-level analyses and the meta-analysis were performed using R software [34] using the *survival*, *meta* and *forestploter* libraries.

## Protocol implementation

The protocol was applied as specified to all available datasets of the participant countries, with one exception. During the early Omicron BA.1 outbreak in Denmark, data transfer of non-Omicron PCR test results was deprioritised from some hospital laboratories, thus, for some hospitalised cases it was only known that they had a non-Omicron variant. Since the predominant non-Omicron variant during this period was Delta, cases infected by non-Omicron variants were assumed to be Delta cases. In a sensitivity analysis, the analysis was restricted to only include the protocol-compliant Delta cases in Denmark whose Delta variant infection had been reported (and those only known to have a non-Omicron variant were excluded).

Outcomes due to any cause were considered as primary outcomes and COVID-19-specific events as secondary outcomes in the protocol. However, because not all participating countries were able to access data on all-cause events, the meta-analysis deviated from the protocol by instead using combined outcomes where HRs of COVID-19-specific events were used when available, and otherwise HRs of all-cause events.

The protocol specified additional subgroup analyses by two variables to estimate vaccination and reinfection status specific and vaccination status and age group specific adjusted HRs for Omicron BA.1 vs Delta. However, only the largest included country (England) had sufficient numbers in all subgroups to reliably estimate these HRs. The results of these subgroup analyses were therefore not included.

## Data analysis pilot

The results of the first round of the applied analysis resulted in further revisions. The analyses by age group or vaccination status had convergence issues when applied to data from some countries with small case numbers. The age groups and vaccination status groups were therefore redefined to ensure sufficient numbers in each category. Vaccination status was further revised to account for the potential effect of waning immunity, by including a separate category for cases who had received two vaccine doses, but the second dose was received more than 5 months (≥ 153 days) before the positive test. We also noticed that for countries with smaller datasets the number of regions to use in adjustments needed be reduced. We recommended that up to approximately five regions were used for countries with few cases or not to use subnational regions for geographically small countries. Guidance was provided on how to select the inclusion period: further instructions were circulated, ensuring that data on both Delta and Omicron cases were available each calendar week. Finally, minor code errors were identified and resolved, and code was added to perform additional checking of input data.

### Omicron BA.1 versus Delta variant severity

After the revision of the protocol and analysis code, the countries re-analysed their datasets using the final updated code (Figure 1B).

A detailed description of each country's data, including COVID-19 testing, variant characterisation methods and data linkages, is available in Supplement 2. Supplement 2 also shows inclusion summaries in Supplementary Figures S1-S6, a summary of available outcomes and adjustment variables in Supplementary Table S1 and descriptive frequencies of outcome events and case characteristics by country in Supplementary Tables S2 and S3, respectively.

Five of the six countries (Denmark, England, Luxembourg, Norway and Scotland) analysed data on cases diagnosed at any age, with mean age ranging between 30.5 and 33.8 years by country. One country (Portugal) included only cases aged ≥ 16 years, with mean age of 40.7 years. Two countries (Denmark and Luxembourg) included only data from cases with virus variants characterised by whole genome sequencing or RT-qPCR, while four countries (England, Norway, Portugal and Scotland) additionally included cases with variants characterised by S gene target surveillance. The inclusion periods differed somewhat between countries due to differences in when Omicron superseded Delta as the dominant circulating variant, in line with the study protocol. The proportion of unvaccinated cases ranged from 11% (1,723/15,619; Portugal) to 39% (1,755/4,453; Luxembourg), and the proportion that had received a booster vaccine dose ranged from 2.7% (5,029/188,405; Denmark) to 22% (18,410/83,278; Scotland), partially reflecting the different inclusion periods.

A total of 1,799 (1.0%) of the 188,405 confirmed COVID-19 cases were hospitalised in Denmark, 16,808 (1.3%) of 1,344,182 in England, 156 (3.5%) of 4,453 in Luxembourg, 610 (0.7%) of 92,918 in Norway, 154 (1.0%) of 15,619 in Portugal and 523 (0.63%) of 83,278 in Scotland. Data on ICU admission were available from four countries: 191 (0.1%) cases in Denmark were treated in ICU, 14 (0.3%) in Luxembourg, 161 (0.2%) in Norway and 115 (0.1%) in Scotland; England and Portugal did not report ICU admission data. All countries had data on the number of deaths: 277 (0.1%) COVID-19 cases died in Denmark, 2,332 (0.2%) in England, 24 (0.5%) in Luxembourg, 122 (0.1%) in Norway, 24 (0.2%) in Portugal and 79 (0.1%) in Scotland. However, in Portugal, no deaths were observed among Omicron BA.1 cases and Portugal was therefore not included in the mortality analysis.

The required and highly desired adjustment variables were available for all participating countries. Of the desired adjustment variables data on ethnicity/country of birth were available for Denmark, England, Norway, Portugal and Scotland, socioeconomic status/deprivation indicator for England, Norway and Scotland, comorbidity for Denmark and Norway and international travel within 14 days before positive test for Luxembourg. Full details on the adjustment variables are available in Supplementary Table S1 in Supplement 2.

### Hazard ratios

**Figure 2:**
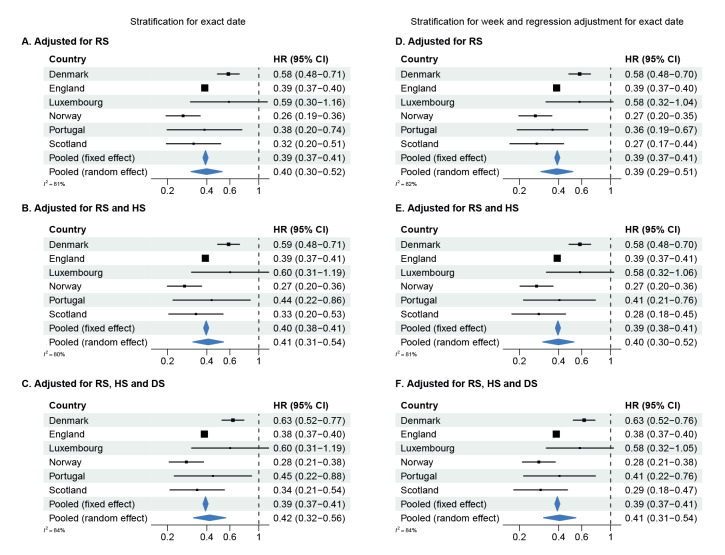
Hazard ratios of hospital admission^a^ for COVID-19 cases infected with the Omicron BA.1 versus Delta variants, by country and pooled across countries using a fixed effect and a random effect model, in six European countries, October 2021–February 2022

CI: confidence interval; DS: desired set of adjustment variables; HR: hazard ratio; HS: highly desired set of adjustment variables; *I* ^2^: proportion of the pooled hazard ratio variance that is attributable to between-country heterogeneity; RS: required set of adjustment variables.

^a^ COVID-19 specific, where available, or otherwise due to any cause.

**Figure 3:**
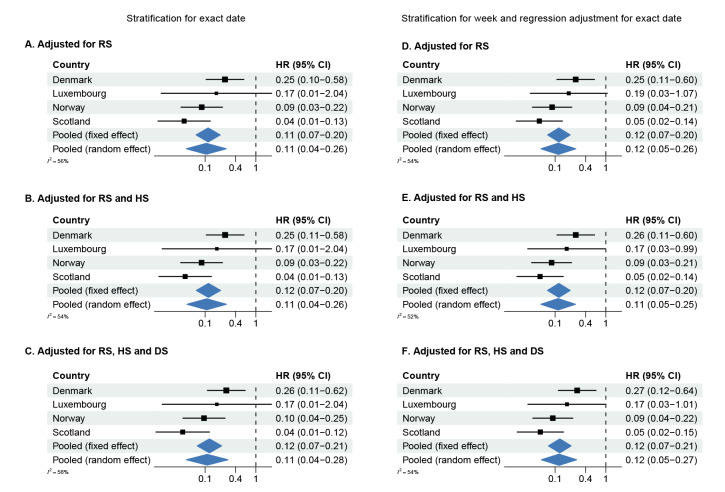
Hazard ratios of admission to intensive care unit^a^ for COVID-19 cases infected with the Omicron BA.1 versus Delta variants, by country and pooled across countries using a fixed effect and a random effect model, in six^b^ European countries, October 2021–February 2022

CI: confidence interval; DS: desired set of adjustment variables; HR: hazard ratio; HS: highly desired set of adjustment variables; *I* ^2^: proportion of the pooled hazard ratio variance that is attributable to between-country heterogeneity; RS: required set of adjustment variables.

^a^ COVID-19 specific.

^b^ England and Portugal did not report data on intensive care unit admissions.

**Figure 4:**
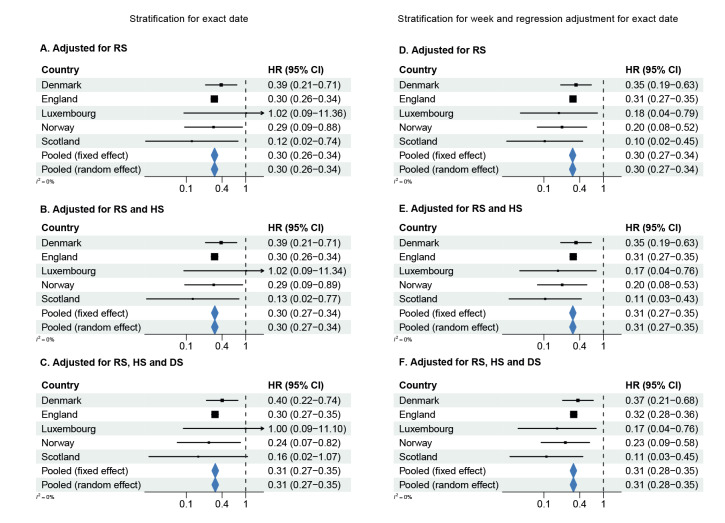
Hazard ratios of death^a^ for COVID-19 cases infected with the Omicron BA.1 versus Delta variants, by country and pooled across countries using a fixed effect and a random effect model, in six^b^ European countries, October 2021–February 2022

CI: confidence interval; DS: desired set of adjustment variables; HR: hazard ratio; HS: highly desired set of adjustment variables; *I* ^2^: proportion of the pooled hazard ratio variance that is attributable to between-country heterogeneity; RS: required set of adjustment variables.

^a^ COVID-19-specific, where available, or otherwise due to any cause.

^b^ In Portugal, no deaths were observed in Omicron BA.1 cases; Portugal’s results were therefore not included in the mortality analysis.

Figures 2, 3, 4 show forest plots of adjusted HRs of hospital admission, ICU admission or death, based on the two adjustment strategies. The corresponding unadjusted HRs are presented in Supplementary Figure S7 in Supplement 2. The HRs from all countries indicated lower risks of all severity outcomes included for cases infected with Omicron BA.1 compared with Delta, both unadjusted and after adjustment for the required variables. Adjustment for the extended set of highly desired or desired adjustment variables changed the HRs only marginally compared with the HRs adjusted for the required adjustment variables only. The pooled random-effects HRs adjusted for the maximum number of adjustment variables were 0.41 (95% confidence interval (CI): 0.31−0.54) for hospital admission, 0.12 (95% CI: 0.05−0.27) for ICU admission, and 0.31 (95% CI: 0.28−0.35) for death.

Adjustment variables: the required adjustment variables were sex, age at diagnosis, date of positive test, area of residence and vaccination status (all available for all countries); the highly desired adjustment variable was reinfection status (available for all countries); the desired adjustment variables were ethnicity/country of birth (available for Denmark, England, Norway, Portugal, Scotland), socioeconomic status/deprivation indicator (available for England, Norway, Scotland), comorbidity (available for Denmark, Norway), and international travel within 14 days before positive test (available for Luxembourg). Full details on the adjustment variables are available in Supplementary Table S1 in Supplement 2.

Adjustment variables: the required adjustment variables were sex, age at diagnosis, date of positive test, area of residence and vaccination status (all available for all countries); the highly desired adjustment variable was reinfection status (available for all countries); the desired adjustment variables were ethnicity/country of birth (available for Denmark, Norway, Scotland), socioeconomic status/deprivation indicator (available for Norway, Scotland), comorbidity (available for Denmark, Norway), and international travel within 14 days before positive test (available for Luxembourg). Full details on the adjustment variables are available in Supplementary Table S1 in Supplement 2.

Adjustment variables: the required adjustment variables were sex, age at diagnosis, date of positive test, area of residence and vaccination status (all available for all countries); the highly desired adjustment variable was reinfection status (available for all countries); the desired adjustment variables were ethnicity/country of birth (available for Denmark, England, Norway, Scotland), socioeconomic status/deprivation indicator (available for England, Norway, Scotland), comorbidity (available for Denmark, Norway), and international travel within 14 days before positive test (available for Luxembourg). Full details on the adjustment variables are available in Supplementary Table S1 in Supplement 2.

**Figure 5:**
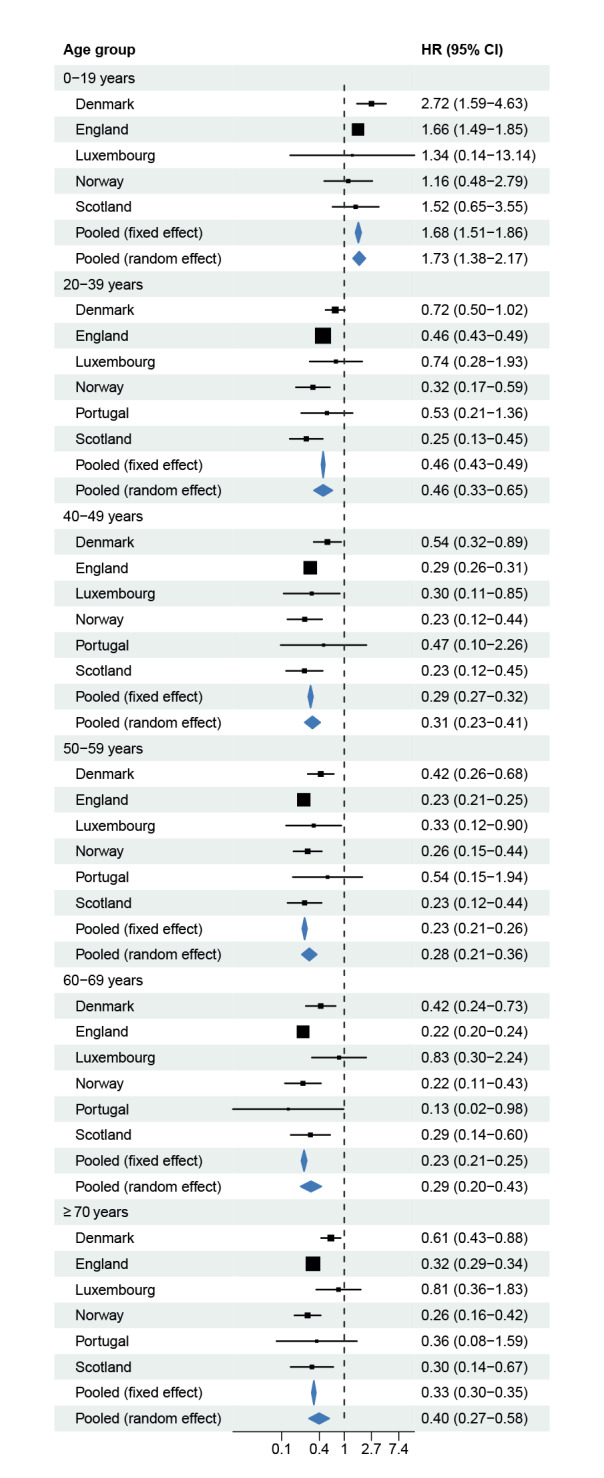
Hazard ratios of hospital admission^a^ for COVID-19 cases infected with the Omicron BA.1 versus Delta variants, by age group, in six European countries, October 2021–February 2022

CI: confidence interval; HR: hazard ratio.

^a^ COVID-19 specific, where available, or otherwise due to any cause.

In subgroups by age (Figure 5), the HRs of hospital admission indicated greater reductions in risk between adult cases infected with Omicron vs Delta than those for children and adolescents. The corresponding HRs of ICU admission or death by age group are shown in Supplementary Figures S8-S9 of Supplement 2.

Adjusted for the required, highly desired and desired set of adjustment variables, using stratification for calendar week and regression adjustment for exact calendar date.

Adjustment variables: the required adjustment variables were sex, age at diagnosis, date of positive test, area of residence and vaccination status (all available for all countries); the highly desired adjustment variable was reinfection status (available for all countries); the desired adjustment variables were ethnicity/country of birth (available for Denmark, England, Norway, Portugal, Scotland), socioeconomic status/deprivation indicator (available for England, Norway, Scotland), comorbidity (available for Denmark, Norway), and international travel within 14 days before positive test (available for Luxembourg). Full details on the adjustment variables are available in Supplementary Table S1 in Supplement 2.

In Portugal, no hospital admissions were observed in the 0–19 years age group; Portugal's results are therefore not included in that sub-analysis.

**Figure 6:**
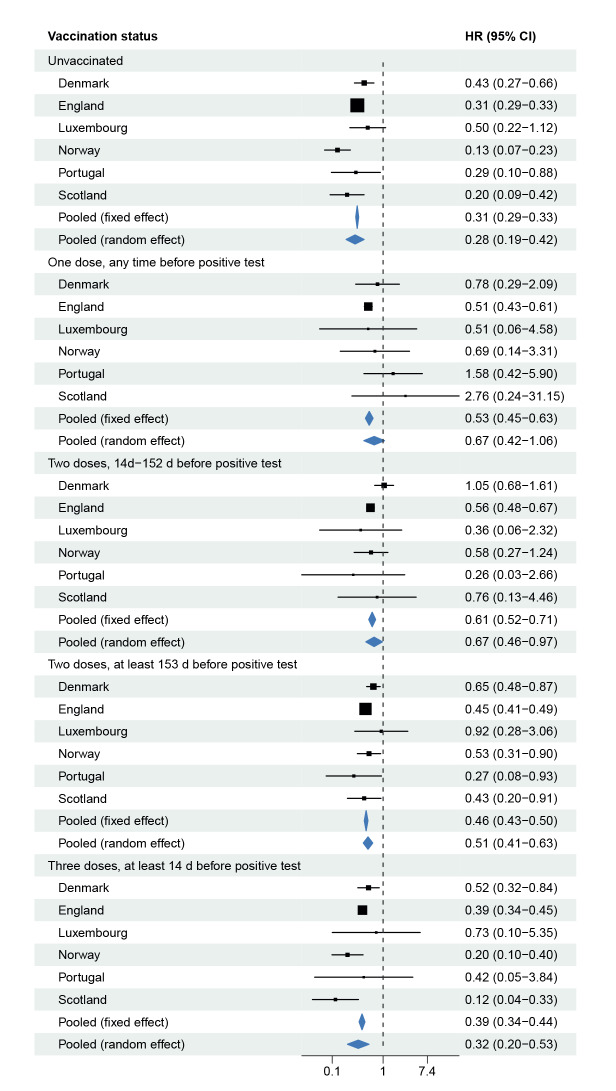
Hazard ratios of hospital admission^a^ for COVID-19 cases infected with the Omicron BA.1 versus Delta variants, by vaccination status in six European countries, October 2021–February 2022

CI: confidence interval; HR: hazard ratio.

^a^ COVID-19-specific, where available, or otherwise due to any cause.

Split by vaccination status (Figure 6), the results indicated lower risks for Omicron compared with Delta cases in all subgroups. The estimated risks were similarly lower for Omicron compared with Delta for cases who were unvaccinated and for cases who had received three vaccine doses. For those who had received one or two vaccine doses, the HRs were somewhat closer to 1. The corresponding HRs of ICU admission or death by vaccination status are shown in Supplementary Figures S10-S11 of Supplement 2.

Adjusted for the required, highly desired and desired set of adjustment variables, using stratification for calendar week and regression adjustment for exact calendar date.

Adjustment variables: the required adjustment variables were sex, age at diagnosis, date of positive test, area of residence and vaccination status (all available for all countries); the highly desired adjustment variable was reinfection status (available for all countries); the desired adjustment variables were ethnicity/country of birth (available for Denmark, England, Norway, Portugal, Scotland), socioeconomic status/deprivation indicator (available for England, Norway, Scotland), comorbidity (available for Denmark, Norway), and international travel within 14 days before positive test (available for Luxembourg). Full details on the adjustment variables are available in Supplementary Table S1 in Supplement 2.

A sensitivity analysis to assess the impact of epidemic phase bias [33], shown in Supplementary Figure S12 in Supplement 2, suggested that the true HRs from all countries might be slightly lower than those estimated, i.e. indicating a slightly larger difference in risk between Omicron and Delta. A sensitivity analysis that excluded the subset of Danish cases assumed in the main analysis to have been infected with Delta due to having their variant characterised as a non-Omicron variant, shown in Supplementary Figure S13 of Supplement 2, found HRs closer to 1 than in the main analysis where all likely Delta cases were included. This is consistent with the assumption that the fraction of hospitalised cases without available data on non-Omicron variants in Denmark were predominantly Delta cases.

The heterogeneity between the adjusted HR estimates was high for the hospital admission (*I* ^2^ = 84%, based on six reporting countries) and ICU admission (*I* ^2^ = 54%, based on four reporting countries) outcomes, but not for death (*I* ^2^ = 0%, based on five reporting countries).

As detailed in Supplement 3, a power calculation indicates that a new variant associated with 50% higher risk relative to a reference variant could be detected with power ≥ 80% using a collaborative meta-analysis between five countries that each analyse national cohort data on 5,000 cases with each variant, if the between-country heterogeneity is at most moderate (*I* ^2^≤50%), or 10,000 cases with each variant if the heterogeneity is high (*I* ^2^=80%). The estimated power is increased if the number of countries is higher, but decreased if the difference in risk is lower.

## Discussion

This is, to our knowledge, the first study to have combined data on the full national cohorts of COVID-19 cases available to public health agencies in several countries to estimate the relative severity of infections with different SARS-CoV-2 variants. This collaborative and standardised effort allowed for the comparison of estimates from several countries and provided pooled estimates with higher precision of the HRs of severe disease outcomes than in analysis of single-country cohorts.

After adjustment for confounders, the pooled results indicate a 59% lower risk of hospital admission, 88% lower risk of ICU admission and 69% lower risk of death for COVID-19 cases infected with Omicron BA.1 relative to those infected with the Delta variant. These lower risks are consistent with estimates reported from previous single-country analyses [1-9].

The results are also consistent with earlier reports that the lower risk for cases with Omicron BA.1 compared with Delta was more pronounced in adults than in children and adolescents [1,3,5,7,35], although some studies have reported conflicting results suggesting a similar risk reduction for children and adults infected with Omicron [2,36]. For cases aged ≥ 20 years, the risk of hospital admission by age group was 54–72% lower for Omicron BA.1 compared with Delta cases. For cases younger than 20 years, the results indicated higher hospitalisation risks for those infected with Omicron BA.1 than Delta. However, this should be considered in the context that absolute risks of severe COVID-19 outcomes are generally low for children and adolescents regardless of the infecting variant [1,3,5,7,35]. Moreover, previous analyses of data from some participant countries have suggested that differences in risk for persons aged < 20 years were considerably smaller when age groups were defined based on narrower age intervals than those considered in this study [1,3,5]. The smaller differences in risks among children could be driven by a different symptom profile of the disease caused by Omicron [3,19,20,37]. Infection with Omicron BA.1 has a higher propensity to cause symptoms in the upper respiratory tract than in the lungs [21,22], which might lead to a greater proportion of young children being admitted on cautionary grounds because of small airways obstruction.

The results indicate ca 70% lower risks of hospitalisation for unvaccinated Omicron BA.1 cases compared with unvaccinated Delta cases and for booster-vaccinated Omicron BA.1 compared with Delta cases who had received a booster dose, but smaller differences in risk (33–49%) between variants for cases who had received one or two vaccine doses. This is consistent with reports of lower vaccine effectiveness against hospitalisation for Omicron BA.1 breakthrough cases who had received only one or two vaccine doses than for corresponding Delta breakthrough cases [3,5,25,26]. Such lower vaccine effectiveness for Omicron BA.1 should be expected to lead to relatively smaller differences in risk between Omicron BA.1 and Delta in these vaccination subgroups, consistent with that observed.

There was high heterogeneity between the HR estimates from the individual countries for hospital and ICU admission but not for mortality. Heterogeneity between national estimates may be expected, and this underlines the added value of comparing and pooling estimates generated via a common protocol to obtain more reliable results for public health decision-making. The heterogeneity might in part reflect differences between countries in definitions for the hospital and ICU admission outcomes. For example, some of the countries considered only COVID-19-related admissions whereas others included all admissions. Some country-specific hospital admission definitions restricted to hospital attendances lasting at least one night whereas other included any recorded admissions, also those that lasted less than one day. By contrast, mortality definitions are unlikely to differ substantially between countries, consistent with the lack of heterogeneity in the HRs of this outcome. Moreover, the heterogeneity was lower between the countries’ hospital admission HRs within subgroups by age or vaccination status, indicating that the heterogeneity may also in part be explained by differences in policies, like testing patterns by age group and vaccination schedules.

In a previous multinational analysis on the severity of illness caused by the Alpha (Pango lineage designation B.1.1.7), Beta (Pango lineage designation B.1.351) and Gamma variants (Pango lineage designation P.1) in seven European countries using a pooled dataset, relatively small numbers of cases with characterised variants were included (range 13 to 9,740 cases/country) [16]. By contrast, the protocol and analysis proposed in our study enables investigators in individual countries to analyse their own national datasets and also allows for multinational collaborations to compare and meta-analyse results from the consistently analysed national datasets, as demonstrated in the Omicron BA.1 vs Delta pilot.

Currently, comprehensive community testing for COVID-19 has been reduced in many countries [27-30], which is likely to pose challenges for estimation of the severity of the illness caused by new virus variants. The protocol is applicable to settings where data are available on a cohort of community cases and is directly applicable in settings with continued community testing or in future pandemic scenarios with widespread community testing. Our power calculation indicates that five countries, each with data on 5,000 cases with each variant if the between-country heterogeneity in the HR estimates is at most moderate, or 10,000 cases with each variant if the heterogeneity is high, may provide sufficient data to detect a clinically relevant increased severity for a new variant. In March 2023, 10 countries in Europe reported sequencing SARS-CoV-2 samples of > 2,000 cases, of which six countries sequenced > 4,000 positive samples (GISAID, https://gisaid.org/). Assuming equal sampling frequency during the month, a meta-analysis using a decentralised approach to pool results from national analyses as proposed here could hence effectively be informed by ca 22,000 sequenced cases/week if applied to these 10 countries, or ca 19,000 cases/week if restricted to the six countries with the highest sequencing frequencies. However, assuming that cases hospitalised due to COVID-19 can be identified (for example via PCR testing at admission of patients with respiratory symptoms) and their infecting variant characterised, case-control type studies may be considered in the absence of wide-reaching community testing. Such a case-control study design could compare the prevalence of different variants between COVID-19 patients who are hospitalised and a suitably chosen comparison group of COVID-19 patients with less severe disease, e.g. cases identified through sentinel community surveillance. However, prevalence of a newly introduced variant generally changes with calendar time and hence the observed prevalence in a sample of COVID-19 cases will depend on their dates of infection. Therefore, care must be taken in the design to ensure that the two groups are comparable in terms of calendar time and the time from infection until testing. A preliminary analysis of the data from England suggests that similar estimates of the RR as from the meta-analysis could be obtained by re-analysing the cohort data using a matched case-control design, provided that the mean time from when hospitalised cases would have tested positive in the community to their hospital admission is known, but that matching hospitalised cases on hospital admission date to community cases on community test date could lead to considerable bias. Further research should explore the feasibility of case-control type designs for monitoring of SARS-CoV-2 variant severity, in single countries and in international collaborations, including ‘right-sizing’ of the optimal rate of community sampling that is required to enable reliable case-severity estimates.

Limitations of the study include a reliance on cases with information on the infecting variant, which might lead to selection bias if there were differences in testing patterns and variant characterisation capacity and capability e.g. by time and locality. However, all the included countries employed community testing for COVID-19 throughout the entire study period. Moreover, the use of stratification for calendar date and geographical region in the data analysis, so that only cases identified under similar testing schedules are compared, should limit the impact of such bias. The inclusion is furthermore complicated by challenges in characterising variants from specimens with low viral load. Omicron BA.1 cases have on average lower viral loads compared with Delta cases [23,24], which might cause collider bias if viral load is also associated with severity [38]. Such bias would, however, likely lead to underestimation of the difference in risk between variants, due to inclusion of Omicron BA.1 case subsets with relatively high viral load. The protocol outlined several potential confounders as adjustment variables. Although all countries could adjust for the most likely confounders in the required and highly desired sets of adjustment variables, no country had the data to adjust for all adjustment variables classified as desired. However, the results from all countries indicated small differences between the HRs adjusted for any available variables additional to the adjustment variables classified as required, which might suggest that the magnitude of confounding due to the expanded set of adjustment variables is small. Age groups were defined with broader age bands than used in some previous single-country analyses [1,3], and the outcome definitions were allowed to differ between countries. This was to accommodate the level of detail available for each country’s data, to enable wider participation. As a result, the outcomes included both COVID-19-specific and all-cause outcomes. For the all-cause outcomes, incidental non-COVID-19-related events are likely to result in a small attenuation of the HRs compared with if COVID-19-specific outcomes had been available. Although this to our knowledge is the largest international collaborative study on Omicron BA.1 vs Delta severity to date, our pilot included no more than six countries. One country, England, had considerably higher case numbers than the other included countries. Therefore, as expected, the HRs from England had great weight on the pooled estimates, particularly the fixed-effect estimates. This might limit the generalisability of the pooled estimates, especially in light of the high heterogeneity of the HRs between countries. However, this highlights the added value of contrasting the country estimates and exploring their heterogeneity.

## Conclusions

This pilot has demonstrated the feasibility of conducting variant case-severity analyses in a multinational collaborative framework. The consistency in the direction of the HR estimates from the real-world analysis, and the high-precision pooled estimates add to the evidence for the relatively lower severity of the illness caused by Omicron BA.1 relative to the Delta variant. The proposed study protocol and accompanying statistical analysis code can facilitate future studies on relative severity by investigators in countries with community testing and available test, virus variant and severe outcome data, which will support public health risk assessments both for future SARS-CoV-2 virus variants and other similar infections.

## Supplementary Data

338000 Supplementary Material 1

## Supplementary Data

779000 Supplementary Material 2

## Supplementary Data

209000 Supplementary Material 3
