# Supplementary material for: A standardised protocol for relative SARS-CoV-2 variant severity assessment, applied to Omicron BA.1 and Delta in six European countries, October 2021 to February 2022
Source: Euro Surveill. 2023 Sep 7;28(36):2300048. doi: 10.2807/1560-7917.ES.2023.28.36.2300048 (PMC10486193; doi:10.2807/1560-7917.ES.2023.28.36.2300048)
Supplement: Supplementary Material 1 [file 23-00048_NYBERG_Supplement1.pdf]

Supplement 1

Study protocol

This supplementary material is hosted by *Eurosurveillance* as supporting information alongside the article “A standardised protocol for relative SARS-CoV-2 variant severity assessment, applied to Omicron BA.1 and Delta in six European countries, October 2021 to February 2022”, on behalf of the authors, who remain responsible for the accuracy and appropriateness of the content. The same standards for ethics, copyright, attributions and permissions as for the article apply. Supplements are not edited by *Eurosurveillance* and the journal is not responsible for the maintenance of any links or email addresses provided therein.

PROTOCOL: Estimating relative case-severity risks by variant

Introduction

During the COVID-19 pandemic of 2020-2022, several new variants of the SARS-CoV-2 virus have evolved that have varied in severity. Several European countries monitor the prevalence of newly evolved or introduced variants ("new" variants) in incident COVID-19 cases and their outcomes, but due to limited numbers these efforts may have only allowed detection of moderate to large differences in severity in individual countries. To enable timely assessment of the relative severity of new variants compared to previously prevalent variants ("old" variants), this protocol proposes (a) a standardised approach to quantifying the relative risks of severe outcomes by variant; and (b) a collaborative effort to pool the results from assessments of relative variant severity between nations, borrowing strength across countries through a meta-analytic approach.

Study Design

National cohort studies of test-positive cases analysed by local investigators, according to a standardised analysis plan outlined below. Statistical summary results (such as relative risk estimates) are collated from each participating country to be synthesised using meta-analysis methods. No individual-level data are transferred outside the participating countries.

The aim of the study described in this protocol is to assess the strength of association between SARS-CoV-2 variant and one or more severity outcomes. The protocol assumes that these outcomes are censored time-to-event type outcomes that may be analysed using survival analysis methods. Such data are expected from participants in the proposed collaborative study. The outlined approach is, however, applicable for studies on severe outcomes of other data types, e.g. binary outcomes, ordinal outcomes, count outcomes, or quantitative outcomes.

Setting

Community testing for SARS-CoV-2 infection in participating European countries.

Participants and Eligibility Criteria

Individual-level data on test-positive COVID-19 cases with available data on SARS-CoV-2 variant, called through whole genome sequencing, genotyping or proxy tests such as assessment of S gene positivity of PCR test specimen. The inclusion calendar period should be chosen as a consecutive period of dates when cases of both variants under study are present. The choice of inclusion period is however left to the discretion of the national study groups.

If the data include cases whose positive specimen have only been assessed via proxy variant tests such as S gene positivity, it is recommended that the inclusion period is restricted to calendar dates when the positive (PPV) and negative predictive values (NPV) of the proxy test to distinguish variants are >90%. PPV is here defined as the probability that cases called as having the new variant with the proxy test do have the new variant based on sequencing-confirmed variant calls, and NPV is defined as the probability that cases called as having the old variant with the proxy test do have the old variant based on sequencing-confirmed variant calls. PPV and NPV generally depend on the prevalence of the new and old variant among new test-positive cases. The calendar-date-specific PPV and NPV can be estimated based on subgroups of cases whose variant has been called both through sequencing and the proxy test.

Exclusion criteria:

- No data on SARS-CoV-2 variant.
- Sequencing- or genotyping-confirmed SARS-CoV-2 variants other than those under study (e.g. rare variants without widespread transmission in the community).
- Data linkage errors (e.g. missing ID number, failure to link with data on outcome or required confounder variables).
- Missing data on at least one of the <u>required</u> confounder variables.
- Vaccination patterns that may indicate immunosuppression:
    - ≥4 vaccination doses received, or
    - vaccination dose 3 received <80 days after dose 2.
- Other data inconsistencies (e.g. likely record errors). These should be specified by each national study group that reports cases excluded on these grounds.

Documentation of ethical approval for use of the individual-level data for research purposes must be provided by each participating national study group.

Variables

Exposure

- SARS-CoV-2 variant or variant sublineage, for example Omicron BA.2 vs Omicron BA.1. Determined based on (in order of precedence):
    a. Whole genome sequencing
    b. Genotyping (if applicable to distinguish lineages/sublineages under study)
    c. S gene positivity or other proxy methods, during calendar periods when calendar-time-specific positive and negative predictive values to distinguish the lineages/sublineages under study is >90%.

Outcomes

In general, the protocol may be followed to study association with any severity outcomes for which there are individual-level data available.

For the proposed collaborative study, the primary outcomes are chosen to be events (hospitalisations, ICU admissions or deaths) due to any cause, and secondary outcomes are chosen to be COVID-19-specific events. The reason for this choice is twofold: (1) events that require no additional data to classify their cause are more likely to be available than data on COVID-19-specific events in all participating countries, and (2) it is a methodological challenge to classify if events are due to COVID-19 and so it would be difficult to standardise a classification that is possible to apply with the data available in different countries.

Collaborative study, primary outcomes

1. Any hospital attendance or admission (any cause; including emergency care attendance or admission through emergency care) within 0-14 days after positive test.
2. Hospital admission (any cause; including admission through emergency care) within 0-14 days after positive test.
3. Admission to intensive care (any cause) within 0-14 days after positive test.
4. Death (any cause) within 0-28 days after positive test.

Collaborative study, secondary outcomes (if available)

5. Any hospital attendance or admission (COVID-19-specific; including emergency care attendance or admission through emergency care) within 0-14 days after positive test.
6. Hospital admission (COVID-19-specific; including admission through emergency care) within 0-14 days after positive test.
7. Admission to intensive care (COVID-19-specific) within 0-14 days after positive test.
8. Death (COVID-19-specific) within 0-28 days after positive test.

The classification of which events are COVID-19-specific will not be standardised and is allowed to differ between countries. The classification from each national study group should be reported to the study coordinators. For example, COVID-19-specific events could be defined as: hospital admissions with COVID-19 specific ICD10 codes; or deaths with COVID-19 as one of the causes of death on the death registration.

For the death outcomes (outcomes 4 and 8), the event-specific follow-up time should be the time from specimen to death if within 28 days, or otherwise the time from specimen to the earliest of the censoring time points (1) the date of latest follow-up/data extraction or (2) 28 days after specimen date. For the hospitalisation/intensive care outcomes (outcomes 1-3 & 5-7), the event-specific follow-up time should be the time from specimen to the event, if within 14 days after specimen, or otherwise the time from specimen to the earliest of the censoring time points (1) date of death, (2) date of latest follow-up/data extraction or (3) 14 days after specimen date. Follow-up times of 0 days (e.g. events on the same day as positive test) should be reset to 0.5 days.

Model and confounder variables

The association between variant (or variant sublineage) and severity outcomes may be confounded by other variables by which the risk of the outcomes varies. In principle, the association should only be confounded to the extent that these variables are differentially associated with the variants/variant sublineages. It is therefore suggested that special care is taken to adjust for calendar date, area/region and vaccination status: when a new variant is replacing an old variant, variant prevalence often varies considerably by calendar time and locality. Calendar time and locality may also be associated with severity outcomes, e.g. through differences in time-and-place-specific healthcare burden or practice. Vaccination status may be associated with variant, if the studied variants have different propensities for breakthrough infections (Andrews et al., 2022), and also with the risk of the outcomes, since the available vaccines offer protection against severe disease in breakthrough cases (Collie et al., 2022).

In addition, several variables have been reported to be associated with risk of severe disease, such as age, sex, comorbidity and socioeconomic factors (Khawaja et al., 2020), although these may not in general be expected to be strongly associated with variant (or variant sublineage). Similarly, some variables such as recent international travel may be suspected to be associated with variant in countries where the new variant is (more recently) imported, but not necessarily strongly associated with the outcome(s). If available, adjustment for these variables may be carried out to rule out potential confounding.

For the collaborative study, two sets of adjustment variables are proposed: (1) one required, minimum set of adjustment variables for which format and categories should be standardised; and (2) one set of additional desired adjustment variables where formatting and categories are allowed to differ to a greater extent according to national setting. Data on all required variables are needed to participate in the collaborative study, whereas research groups may participate regardless of available data on the desired variables.

**Variable**	**Description**	**Data type**	**Categorisation (if applicable)**
**Required**			
VOC	SARS-CoV-2 variant or variant sublineage.	Categorical variable.	• [Old variant]<br>• [New variant]
DATE	Calendar date of positive test. Earliest positive test in the most recent infection episode.	Date variable.	
WEEK	ISO calendar week of positive test.	Categorical variable.	Defined based on the DATE variable. Categories are determined by the inclusion period.
AREA*	Area/region of residence.	Categorical variable.	Categories are allowed to differ according to national setting, but are recommended to be chosen so as to align with

			healthcare administrative areas/regions. For geographically small countries, a single region may be used for the entire country.
VACC	Vaccination status at date of positive test.	Categorical variable.	Preferred categorisation:<br>● Unvaccinated<br>● <28 days after first dose<br>● ≥28 days after first dose and <14 days after second dose, if any<br>● 14-152 days after second dose and <14 days after third dose, if any<br>● ≥153 days after second dose and <14 days after third dose, if any<br>● ≥14 days after third dose<br>If the vaccination data available do not allow for the above categorisation, the study groups are asked to contact the study coordinators with a description of the available vaccination data.
AGE	Age on date of positive test (in years).	Numeric variable.	
AGE10YR	Age group (10- or 20-year bands).	Categorical variable.	Defined based on the AGE variable.†<br>● 0-19 years<br>● 20-39 years<br>● 40-49 years<br>● 50-59 years<br>● 60-69 years<br>● ≥70 years
SEX	Biological sex.	Categorical variable.	● Female<br>● Male
**Highly desired**			
REINF	Known reinfection, defined as ≥1 known past positive test >90 days before current infection episode.	Categorical variable.	● No known past infection.<br>● Known past infection.
**Desired**			
ETHN*	Ethnic group, and/or country of birth.	Categorical variable(s).	Categories allowed to differ according to national setting. Include “Unknown” as a category for cases with missing data (if any).
SES*	Socioeconomic status/deprivation indicators.	Categorical variable(s).	Categories allowed to differ according to national setting. Include “Unknown” as a category for cases with missing data (if any).
COMORB*	Comorbidity.	Categorical variable(s).	Charlson Comorbidity Index (preferred), or otherwise allowed to differ according to national setting. Include “Unknown” as a category for cases with missing data (if

			any).
TRAVEL	International travel from any country within 14 days before testing positive.	Categorical variable.	• No international travel within 14 days before testing positive.<br>• International travel within 14 days before testing positive.<br>Include "Unknown" as a category for cases with missing data (if any).

* Categories are allowed to differ according to national setting.

If there are potentially relevant data available on some of the above variables but the available data do not allow for the classification to be in the recommended format, the study groups are asked to please contact the study coordinators to discuss their applicability.

Data sources / linkage

Case- and/or episode-based individual-level data record-linked (if necessary) to outcome, confounder and variant datasets.

Statistical methods

To provide relative risk estimates that are adjusted for potential confounders, it is recommended that the investigators use conditional methods such as stratification for the confounders considered most likely to confound the association a priori. Stratification is a statistical method that is conceptually similar to matching, but where data from all potential matches are used instead of e.g. a fixed number of comparison cases with an old variant for each case with a new variant. Stratified Cox regression can be used where the outcome(s) are censored time-to-event type data, allowing for a different baseline hazard in each stratum. It is readily available in most standard statistical software. Similar methods are available for other types of outcome data. For example, conditional logistic regression may be used for binary outcomes, or conditional Poisson regression may be used for binary or count outcomes, to achieve similar stratification-based adjustment. The study coordinators will share standardised and fully documented R code for stratified Cox regression, and will advise on implementation.

For the collaborative study

A summary of the number of cases included and excluded should be provided by each participating study, in a standardised format provided in the Appendix (Appendix Figure 1). This summary should

provide sufficient information to calculate the proportion of all cases in the country during the inclusion period that were included in the analysis. The same summary figure may be used if the protocol is used in separate studies.

Descriptive frequencies of the distribution of the confounders by the cases' SARS-CoV-2 variant or variant sublineage should be provided from each participating study using the template provided in the Appendix (Appendix Table 1).

Stratified Cox proportional hazards regression should be used to estimate hazard ratios of new vs old variant lineage/sublineage, while adjusting for confounders and accounting for administrative censoring due to not all cases necessarily having a complete follow-up of 14 or 28 days.

Because of the potential association with both exposure and outcome for vaccination status, and since adjustment for calendar-period-and-locality-specific confounders such as healthcare burden is only through calendar date and area information, stratification-based methods should be used to ensure that the analysis is informed only by cases with the same vaccination status, from the same calendar periods and areas. Two alternative stratification approaches should be used: one that prioritises stricter control for exact calendar date (but which may not be feasible for smaller sample sizes); and one that prioritises somewhat higher precision while still matching for calendar week and adjusting for exact calendar date:

- Stratification for vaccination status, area and exact calendar date of specimen.
- Stratification for vaccination status, area and ISO calendar week of specimen, and including an interaction term between calendar week and linear calendar date.

Primary analyses

The following primary models should be fitted. Note that missing data on any of the included required variables is an exclusion criterion.

Model 1a. The primary model should include the effects of:

Stratification for:

- DATE
- AREA
- VACC

Fixed effects regression terms for:

- VOC
- AGE10YR
- AGE10YR × AGE (interaction term)
- SEX

Model 1b. A model with the same variables as in Model 1a, but with stratification for exact calendar date replaced by:

Stratification for:

- WEEK
- AREA
- VACC

Fixed effects regression terms for:

- VOC
- AGE10YR
- AGE10YR ⨉ AGE (interaction term)
- SEX
- WEEK ⨉ DATE (interaction term)

Secondary analyses

Secondarily, two more sets of models may be fitted if the investigators have access to data on additional confounders.

Models 2a and 2b. Models including all variables in Models 1a and 1b respectively, with additionally a fixed effects regression term for:

- REINF (if available).

Models 3a and 3b. Models including all variables in Models 1a and 1b respectively, with additionally fixed effects regression terms for all available variables out of:

- REINF (if available).
- ETHN (if available).
- SES (if available).
- COMORB (if available).
- TRAVEL (if available).

If only a subset of the above variables are available, those may be used instead in this secondary model.

Subgroup analyses

- By age groups: Models should be fitted in the same format as Models 1a, 1b, 2a, 2b, 3a and 3b, but with an additional interaction term between variant and age group to estimate age-group-specific HRs.
    - AGE10YR ⨉ VOC (interaction term).

- By vaccination status: Models should be fitted in the same format as Models 1a, 1b, 2a, 2b, 3a and 3b, but with an additional interaction term between variant and vaccination status to estimate vaccination-status-specific HRs.
    - VACC ╳ VOC (interaction term).

- By vaccination status and reinfection status (if data on reinfection status available): Models should be fitted in the same format as Models 2a, 2b, 3a and 3b, but with additional interaction terms between variant, vaccination status and reinfection status to estimate vaccination-and-reinfection-status-specific HRs.
    - VACC ╳ VOC + REINF ╳ VOC + VACC ╳ REINF ╳ VOC (interaction terms).

- By vaccination status and age group: Models should be fitted in the same format as Models 2a, 2b, 3a and 3b, but with additional interaction terms between variant, vaccination status and age group to estimate vaccination-status-and-age-group-specific HRs.
    - VACC ╳ VOC + AGE10YR ╳ VOC + VACC ╳ AGE10YR ╳ VOC (interaction terms).

Sensitivity analyses

The local investigators are recommended to investigate the sensitivity of the hazard ratios to the variant classification methods, particularly as sequencing may be biased towards more severe/hospitalised cases, although this potential bias is not expected to vary by variant. Furthermore, relative severity estimates may be affected by so-called epidemic phase bias (Seaman et al., 2022). Epidemic phase bias may occur when comparing two variants, if: (1) date of positive test is used to define inclusion period and/or is an adjustment variable; (2) the incidence of one variant is growing and the incidence of the other variant is declining, or where the incidence of one variant is growing faster than the incidence of the other variant; and (3) there is a correlation between the time from infection to positive test and disease severity. In such situations, the observed risk associated with the faster growing variant may be overestimated compared to the observed risk associated with the declining/slower growing variant (Seaman et al., 2022).

- Sensitivity to variant classification: repeat Models 1a and 1b, restricted to cases whose variant or variant sublineage was called by each of the used variant calling methods, e.g. sequencing (if available), genotyping (if available), or proxy tests such as S gene positivity (if available).

- Epidemic phase bias: A sensitivity analysis to investigate the impact of epidemic phase bias under different assumed scenarios was recently proposed, that involves refitting models to a calendar date that is shifted for cases who experience severe outcomes, according to an assumed difference between the mean time from infection to positive test in cases who do not experience severe outcomes and the mean time from infection to positive test in cases who do experience severe outcomes (Seaman et al., 2022). It is recommended that the

models are repeated under a range of scenarios with assumed differences ranging from 1 to 4 days. Code and detailed guidance can be provided by the study coordinators.

Further sensitivity analyses should be considered by local investigators, although they are not required to take part in the collaborative study synthesising estimates. These further assessments include:

- Sensitivity to adjustment strategy:
  - vary the number of adjustment variables and/or changing which variables are stratification or regression variables.

- Sensitivity to outcome definition(s):
  - modify the time period within which to consider severe outcomes (e.g. admissions or deaths within X days, varying X);
  - modify the criteria used to classify COVID-19-specific events.

Outputs

For each of Models 1a, 1b, 2a, 2b, 3a and 3b and each sensitivity analysis, standardised code and guidance will be provided by the study coordinators to report the key effect size needed, i.e. the hazard ratio of each outcome for the new variant compared to the old variant.

For each of the subgroup analyses, the new variant:old variant hazard ratio of each outcome will be reported for each subgroup.

Meta-analysis

After receiving the HR estimates and their associated standard errors from each country, the study coordinators will use fixed and random effects meta-analysis to combine the estimates from several countries into pooled HR estimates from each of the models (1a, 1b, 2a, 2b, 3a, 3b) and outcomes, and for each of the subgroup analysis models. The estimates from each country will be contrasted in forest plots and outliers will be investigated using funnel plots. The I^2 statistic will be used to formally quantify the heterogeneity of the estimates between countries.

Strengths and limitations

Strengths of synthesising the results from several countries include the larger effective sample sizes that meta-analysis enables, which may lead to more rapid assessment of the risk associated with new variants. The analyses being run by separate national analysis teams ensures that local

knowledge about the specifics of each country's data collection practices and other sources of bias are retained. Meta-analysis further allows for the results from different countries to be contrasted, which in turn will inform whether observed risk patterns with new variants are consistent between countries. Limitations include the reliance on pre-calculated estimates as opposed to if access and analysis of the individual-level data were centralised. However, the loss of precision of meta-analysis of country-level relative risk estimates compared to analysis of pooled individual-level data are likely acceptably small, and is a reasonable compromise that enables more rapid analysis of variant-specific risks than what international transfer of data would entail.

References

Andrews, N., Stowe, J., Kirsebom, F., Toffa, S., Rickeard, T., Gallagher, E., Gower, C., Kall, M., Groves, N., O'Connell, A.-M., Simons, D., Blomquist, P. B., Zaidi, A., Nash, S., Iwani Binti Abdul Aziz, N., Thelwall, S., Dabrera, G., Myers, R., Amirthalingam, G., . . . Lopez Bernal, J. (2022). Covid-19 Vaccine Effectiveness against the Omicron (B.1.1.529) Variant. *New England Journal of Medicine*, *386*(16), 1532-1546. https://doi.org/10.1056/NEJMoa2119451

Collie, S., Champion, J., Moultrie, H., Bekker, L.-G., & Gray, G. (2022). Effectiveness of BNT162b2 Vaccine against Omicron Variant in South Africa. *New England Journal of Medicine*, *386*(5), 494-496. https://doi.org/10.1056/NEJMc2119270

Khawaja, A. P., Warwick, A. N., Hysi, P. G., Kastner, A., Dick, A., Khaw, P. T., Tufail, A., Foster, P. J., & Khaw, K.-T. (2020). Associations with covid-19 hospitalisation amongst 406,793 adults: the UK Biobank prospective cohort study. *medRxiv*, 2020.2005.2006.20092957. https://doi.org/10.1101/2020.05.06.20092957

Seaman, S. R., Nyberg, T., Overton, C. E., Pascall, D. J., Presanis, A. M., & De Angelis, D. (2022). Adjusting for time of infection or positive test when estimating the risk of a post-infection outcome in an epidemic. *Statistical Methods in Medical Research*, *31*(10), 1942-1958. https://doi.org/10.1177/09622802221107105

APPENDIX Figure 1: Inclusion flow chart

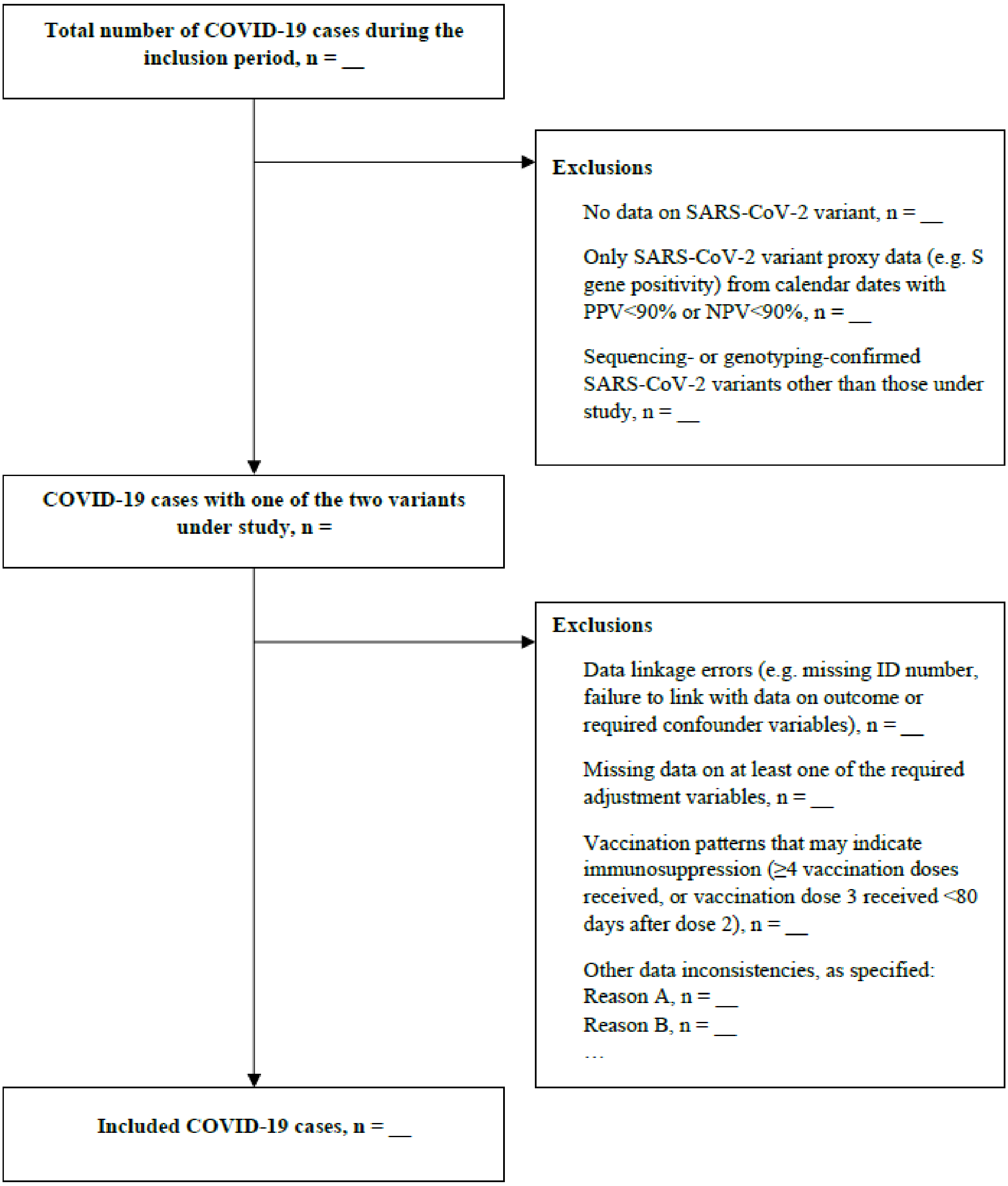

APPENDIX Table 1: Characteristics

Characteristic		Overall	[Old variant]	[New variant]
		**n (%)**	**n (%)**	**n (%)**
Total				
Age				
	0-19			
	20-39			
	40-49			
	50-59			
	60-69			
	≥70			
Sex				
	Female			
	Male			
Calendar week of positive test				
	ISO week w			
	ISO week w+1			
	ISO week w+2			
	…			
Area/region of residence				

	Region A			
	Region B			
	Region C			
	…			
Vaccination status at date of specimen				
	Unvaccinated			
	<28 days after first dose			
	≥28 days after first dose and <14 days after second dose			
	14-152 days after second dose and <14 days after third dose			
	≥153 days after second dose and <14 days after third dose			
	≥14 days after third dose			
Reinfection status				
	First infection episode			
	Reinfection episode			
Ethnicity/country of birth*				
	Ethnicity A			
	Ethnicity B			
	Ethnicity C			
	…			
	Unknown			

Socioeconomic index/deprivation indicator(s)*				
	Category A			
	Category B			
	Category C			
	…			
	Unknown			
Comorbidity*				
	Comorbidity category A			
	Comorbidity category B			
	Comorbidity category C			
	…			
	Unknown			
International travel within 14 days before positive test				
	No international travel			
	International travel			
	Unknown			

* The number of variables and the categories to report for these factors is not standardised and is allowed to vary according to the data available in each local setting.
