# Supplementary material for: A standardised protocol for relative SARS-CoV-2 variant severity assessment, applied to Omicron BA.1 and Delta in six European countries, October 2021 to February 2022
Source: Euro Surveill. 2023 Sep 7;28(36):2300048. doi: 10.2807/1560-7917.ES.2023.28.36.2300048 (PMC10486193; doi:10.2807/1560-7917.ES.2023.28.36.2300048)
Supplement: Supplementary Material 2 [file 23-00048_NYBERG_Supplement2.pdf]

Supplement 2

Pilot to estimate Omicron BA.1 versus Delta variant severity

This supplementary material is hosted by *Eurosurveillance* as supporting information alongside the article “A standardised protocol for relative SARS-CoV-2 variant severity assessment, applied to Omicron BA.1 and Delta in six European countries, October 2021 to February 2022”, on behalf of the authors, who remain responsible for the accuracy and appropriateness of the content. The same standards for ethics, copyright, attributions and permissions as for the article apply. Supplements are not edited by *Eurosurveillance* and the journal is not responsible for the maintenance of any links or email addresses provided therein.

Supplementary text: Country-level data descriptions

Denmark

Data sources and linkage

We obtained data from the National Microbiology Database for all individuals tested with SARS-CoV-2 by RT-PCR in Denmark since March 1, 2020, and data from other national registers, available in the national COVID-19 surveillance system database at Statens Serum Institut (Copenhagen, Denmark), described in detail elsewhere [1]. Briefly, the surveillance system links individual-level information daily between national registers and databases by the use of the unique personal identification number of all Danish citizens, thereby centralising surveillance information from the National Patient Register (inpatient and outpatient diagnoses, admission and discharge dates), the National Vaccination Registry, the Civil Registration System (vital status and previous and current addresses), and variant-specific RT-PCR test results from the National Microbiology Database.

Testing practices

Free-of-charge RT-PCR testing for close contacts of cases, those with mild COVID-19 symptoms, and asymptomatic individuals was provided by a centralized public COVID-19 test laboratory, Test Center Danmark (TCDK). All tests are provided as part of a universal tax-funded health-care system. Test timeslots are publicly available and can be booked online, and public tests sites are densely located across the country and open daily also for walk-in testing.

Individuals with symptoms suggestive of COVID-19 seen by a doctor (along with health-care personnel) were PCR tested in regional clinics and hospitals connected with the ten Danish departments of clinical microbiology (CMDs). CMDs serve both public and private hospitals, primary health clinics and long-term care facilities with suspected or ongoing outbreaks.

Variant classification

Variants of cases analysed were determined using variant-specific RT-PCR testing. In the community track, an RT-PCR test using the L452 marker was developed and implemented by the Test Center Denmark (TCDK) by Dec 1, 2021. The estimated specificity was 99.99% based on retrospective analysis. In the health care track, eight of ten local Clinical Microbiology Departments (CMD) had similar omicron-specific variant-specific RT-PCR solutions set up and documented; the remaining two of ten local CMDs accounted for less than 7% of RT-PCR tests done by CMDs.

For the community track variant RT-PCR by TCDK, a confirmed omicron case was defined as a case testing positive with RT-PCR targeting the wild type sequence L452, as described elsewhere [2]. The delta variant is detected by the 452R substitution and was the only predominant variant in Denmark (>99%) in the period before occurrence of omicron; individuals testing positive for the 452R substitution were therefore considered to be infected with the delta VOC.

Outcome data and definitions

A hospital admission was identified using three different data sources: 1) The National Patient Register which contains any contact to a Danish hospital longer than 12 h, 2) Twice daily hospital data (snapshots) in each region, which also includes admissions of duration less than 12 h on the condition that a bed is assigned to the patient, 3) All samples from hospitalised patients are confirmed as SARS-CoV-2 positive in the National Microbiology Database. COVID-19 specific hospital admission was defined by the main diagnosis of the hospitalisation having ICD10-codes DB342A, DB972A, DB972B, or DB948A. Information on ICU treatment was furthermore obtained from The

National Patient Register. Hospital admission and ICU admission that occurred 0-14 days after positive test was considered.

Data on deaths were obtained from the Danish Civil Registration System and the Cause of Death Register. Deaths due to any cause were considered if they occurred within 0-28 days after positive test.

Ethical approval

The Danish study was performed as a surveillance study as part of the governmental institution Statens Serum Institut's advisory tasks for the Danish Ministry of Health. Statens Serum Institut's purpose is to prevent and fight the spread of infectious diseases in Denmark as specified in section 222 of the Danish Health Care Act. The need for ethics approval and informed consent is therefore deemed unnecessary according to national legislation, cf. implementing decree 2020-09-01, number 1338, about scientific regulatory procedure of health science research projects and health data scientific research projects. The presented data contains aggregated results and no personal data, and is therefore not subject to the European General Data Protection Regulation.

England

Data sources and linkage

COVID-19 is a notifiable disease in England, and all cases who test positive for the disease are required to be reported to the Second Generation Surveillance System (SGSS) at the UK Health Security Agency (UKHSA). This data resource includes baseline characteristics such as age, sex, ethnicity, region of residence, and index of multiple deprivation (an area-level classification of relative deprivation).

Here, data on all cases with positive test dates between 29 November and 30 December 2021 were extracted, and individually linked to variant classification results based on whole genome sequencing, provisional genotyping, or assessment of S gene positivity. These data were further linked to SARS-CoV-2 vaccination status through the National Immunisation Management Service; to hospitalisation data from two sources (Emergency Care Data Set and Secondary Uses Service); and to a mortality line list for COVID-19 cases maintained by UKHSA. All data were extracted and linked on 13 May 2022.

Testing practices

During the inclusion period, community testing was widely available, including cost-free lateral flow testing of asymptomatic individuals in the community, cost-free PCR-based community testing of symptomatic individuals, and targeted routine testing of healthcare staff, hospitalised individuals and international travellers.

Variant classification

During the study period, the variants of confirmed specimens were classified based on whole genome sequencing, provisional genotyping, or S gene positivity. Specimens were selected for whole genome sequencing or provisional genotyping based on geographically weighted population-level sampling of community cases, and targeted selection of hospitalised cases, hospital staff and recent international travellers. This variant classification was supplemented with S gene positivity data for specimens from England's community testing programme. Community test specimens were submitted for assessment with custom TaqPath assays at three laboratories that served the entire country depending on demand. The Delta variant is associated with S gene positivity and the

Omicron BA.1 variant with S gene negativity, but S gene status is not specific to these two variants. Therefore, the inclusion period for the analysis was chosen to ensure a sufficient prevalence of both variants to ensure high predictive values when S gene positivity/negativity was used to classify variants as Delta or Omicron.

Outcome data and definitions

Hospital admissions were extracted from two sources, the Emergency Care Data Set (ECDS) and the Secondary Uses Service (SUS). ECDS includes data on all attendances to emergency care departments in NHS hospitals, including those that result in admission, and is typically updated on a daily basis. SUS includes data on all hospital admissions to NHS hospitals, with reporting mandated at the time of discharge, and is updated monthly. Based on these two complementary data sources, hospitalisation events were classified as admissions within 14 days if the hospitalisation occurred within 0-14 days after positive test and (1) the time between admission and discharge was ≥1 day, (2) ECDS records indicated admission or transfer to another hospital department, or (3) the patient died in hospital on the same day as attendance.

Data on deaths in COVID-19 cases after positive test were collected by the UKHSA from four sources: (1) deaths in hospitals, (2) deaths notified to Health Protection Teams during outbreak management, (3) linkage of COVID-19 tests with death reports, and (4) death registrations where COVID-19 was mentioned on the death certificate. Here, deaths that occurred within 0-28 days due to any cause were considered.

Ethical approval

This surveillance analysis was performed as part of UK Health Security Agency's (UKHSA) responsibility to monitor COVID-19 during the current pandemic. UKHSA has legal permission, provided by Regulation 3 of The Health Service (Control of Patient Information) Regulations 2002 to process confidential patient information under Sections 3(i) (a) to (c), 3(i)(d) (i) and (ii) and 3(iii) as part of its outbreak response activities. This study falls within the research activities approved by the UKHSA Research Ethics and Governance Group.

Luxembourg

Data sources and linkage

COVID-19 case were established from the contact tracing database (CARE+) which automatically receives all lab-confirmed cases. It also includes all antigenic results which are reported from pharmacies or health professionals.

Specific data collection systems were set up for monitoring hospital admissions and deaths from nursing homes and hospitals. Death outcomes were ascertained using death certificates. Vaccination status was obtained from a national vaccination COVID-19 database (MSVAC) set up for that particular purpose. Data bases were linked using a pseudonymised national personal identifier.

Testing practices

PCR testing was widely available and did not change during the period, i.e. both symptomatic and asymptomatic cases could get tested. Testing was free following a medical prescription and for persons who self-declared a positive antigen test. There were twice weekly antigenic screening programmes in primary and secondary schools, as well as monthly screening in long term care facilities.

A twice weekly rapid antigen programme was in place in primary schools and once per week in secondary schools. All positive tests were in principle followed up by a PCR. Antigenic and PCR tests were also conducted in nursing homes when they had a cluster of cases.

Rapid antigen tests were available for purchase at relatively low cost in supermarkets and pharmacies. People were encouraged to test themselves prior to meeting up, e.g. for family get together sets.

Variant classification
The national reference laboratory for acute respiratory infections receives SARS-CoV-2 positive samples (nasopharyngeal or oropharyngeal swabs analysed by RT-PCR) from the national network of laboratories. According to ECDC guidelines a representative sample of specimens chosen through random systemic selection is used for sequencing. Targeted sequencing was also conducted on hospitalised cases, breakthrough infections, or from large transmission clusters, e.g. in nursing homes. Overall, approximately 20% of confirmed cases were sequenced.

Outcome data and definitions
Hospitals had to notify via a specific data collection tool, admissions which could potentially be related to a COVID-19: either suspected cases (e.g. in ICU based on scanner results), PCR test positive cases, and cases who were admitted for non-covid-19 reasons, but were positive at admission screening and who had to be isolated. ICU admissions included all hospitalisation events as above who went to ICU. Deaths were collected as any deaths that occurred within 28 days of the PCR positive test.

Ethical approval
The current analysis is based on pseudonymized surveillance data collected by the Ministry of Health within the framework of the COVID-19 pandemic. The law on COVID-19 (https://legilux.public.lu/eli/etat/leg/loi/2020/07/17/a624/consolide/20210716) explicitly permits scientific research studies on the effects of vaccines against COVID-19 disease subject to prior pseudonymization.

Norway

Data sources and linkage
All data in this study for Norway came from the national emergency preparedness register for COVID-19, Beredt C19 (https://www.fhi.no/en/id/infectious-diseases/coronavirus/emergency-preparedness-register-for-covid-19/). Beredt C19 contains individual-level data from central health registries, national clinical registries and other national administrative registries. It covers all residents in Norway, and includes data on all laboratory-confirmed cases of COVID-19 in Norway, all hospitalizations among cases, deaths, and COVID-19 vaccinations. All databases were linked based on the national identity number.

Testing practices
SARS-CoV-2 tests, both PCR and rapid antigen/lateral flow tests, were available free of charge for everyone during the study period, including those with mild or no symptoms, close contacts, and individuals in quarantine. Anyone who wished to be tested could get tested. Positive rapid antigen/lateral flow tests were recommended to be confirmed with PCR. Patients admitted to hospital were routinely tested for COVID-19. In addition, routine biweekly screening of school children with rapid antigen tests in areas with high transmission was recommended for secondary

school students from late August 2021 and for primary school students from November 2021 until the end of January 2022. From 3 December 2021 until the start of February 2022, all travelers arriving to Norway were required to get tested.

Variant classification

Data on virus variants came from the MSIS laboratory database (national laboratory database), which receives SARS-CoV-2 test results from all Norwegian microbiology laboratories. Variants were identified based on whole genome sequencing, Sanger partial S-gene sequencing or PCR screening targeting specific single nucleotide polymorphisms, insertions or deletions that reliably differentiate between Omicron and other variants. Details on SARS-CoV-2 variant surveillance and classification have been published by the Norwegian Institute of Public Health (in Norwegian): *https://www.fhi.no/nettpub/veileder-for-mikrobiologiske-laboratorieanalyser/covid-19/pavisning-og-overvakning-av-sars-cov-2-virusvarianter/*.

Outcome data and definitions

We obtained data on hospitalisation following a positive SARS-CoV-2 test from the Norwegian Intensive Care and Pandemic Registry (NIPaR, part of Beredt C19) and data on COVID-19 related deaths from the Cause of Death Registry (DÅR, part of Beredt C19).

COVID-19 hospitalisation

In the study period, all Norwegian hospitals reported to NIPaR, and reporting was mandatory. Hospitals in Norway functioned within capacity during the study period, and criteria for hospitalisation and isolation for COVID-19 patients were consistent. During the study period, hospitals were encouraged to register new admissions within 24 hours. Reporting was timely. The median time from admission to registration in the period week 50 2021 – week 1 2022 was 0.9 days (interquartile range: 0.6 – 2.0 days. Hospitalisation was defined as hospital admission following a positive SARS-CoV-2 test, where COVID-19 was reported as the main cause of admission, based on a clinical assessment. Cases hospitalised with other or unknown main cause of admission were excluded from the study population in order to avoid bias. All admissions to hospital, regardless of length of stay, were included. Full details on the registration of patients hospitalised with COVID-19 are available here (in Norwegian): *https://helse-bergen.no/norsk-pandemiregister/registrering-i-norsk-pandemiregister-informasjon-til-ansatte.*

ICU admission

Patients are registered as ICU patients in NIPaR if they fulfil one of five categories:

- Length of stay over 24 hours in intensive care
- Require mechanical ventilation
- Are transferred between intensive care wards
- Persistent administration of vasoactive medication
- Length of stay under 24 hours, but passed away during stay in intensive care

Full details on the registration of ICU patients are available here (in Norwegian): *https://helse-bergen.no/norsk-intensivregister-nir/registrering-av-data-i-nir-kun-for-medlemmer.*

COVID-19 deaths

COVID-19 deaths are defined as deaths reported with COVID-19 as the main cause of death or contributing cause on the death certificate. More details are available here (in Norwegian):

https://www.fhi.no/sv/smittsomme-sykdommer/corona/dags--og-ukerapporter/sporsmal-og-svar-om-koronaovervaking-og-statistikk/.

Ethical approval

Ethical approval for this study in Norway was granted by Regional Committees for Medical Research Ethics - South East Norway, reference number 249509.

Portugal

Data sources and linkage

The study population was individuals eligible for vaccination (≥16 years old) diagnosed with SARS-CoV-2 infection/COVID-19 by nasopharyngeal swab tested with RT-PCR between December 1st and 29th 2021 notified through the laboratory service of the national surveillance system (SINAVE), in Portugal mainland. For this sample we excluded individuals with lateral flow tests. We included individuals with samples classified either by whole-genome sequencing or Spike Gene Target Failure (SGTF). Both symptomatic and asymptomatic individuals were included.

We obtained COVID-19 vaccination status through the electronic national vaccination register (VACINAS). Vaccination status was indexed at the date of COVID-19 diagnosis.

Information about age, sex and date of diagnosis, nationality and area of residency was routinely collected on surveillance system SINAVE and was extracted from it. Previous infection was defined as a PCR or rapid antigen SARS-CoV-2 notification for the same individual more than 90 days apart.

Testing practices

PCR testing is widely available and free of charge, but mainly to symptomatic individuals or to asymptomatic patients in healthcare settings. At the time of the study asymptomatic individuals had access to free of charge lateral flow tests. Most community pharmacies offered lateral flow tests to all the individuals regardless of their symptoms. All patients admitted to the hospital have a mandatory PCR test regardless of the symptoms. Patients in Long Term Care facilities might have routine testing.

Variant classification

We used information from a nationwide network group of laboratories (UNILABS, ABC and CVP) that performs RT-PCR tests for SARS-CoV-2 using Thermofisher TaqPath assay, targeting three regions of the SARS-CoV-2 genome: ORF1ab, N and S genes. Their samples are classified as BA.1 (no amplification) or B.1.617.2 (amplification), according to SGTF status. Only samples having both N and ORF1a positive signals and Ct values ≤30 were considered. Secondly, we use Whole-Genome Sequencing (WGS) data provided by the National Health Institute (INSA) that routinely performs sequencing on random samples notified to SINAVE. INSA follows the European Center for Disease and Control WGS sampling guidelines aiming at a 1% to 3% of total cases with WGS. Variants were classified first by whole genomic sequencing (WGS) and, if this information was unavailable, by detecting the S gene target failure (SGTF). The proportion of whole genomic sequencing (WGS) in the overall samples was 11% for Delta and 4% for Omicron BA.1, we used SGTF in the other samples to identify the variant.

Outcome data and definitions

We defined COVID-19 hospitalization as any admission to a public hospital in Portugal mainland within the 14 days following a positive sample SARS-CoV-2 collection, notified in the surveillance

system, hence the last cases were followed until 12th of January. Admission data was obtained through the Central Hospital Morbidity Database and Integrated Hospital Information System. The Central Hospital Morbidity Database gathers data from all public hospitals in Portugal mainland, which account for most hospitals in Portugal and most hospitals admitting patients with COVID-19. Individuals with a SARS-CoV-2 diagnosis (by sample collection day) or notification after the admission date were excluded.

A COVID-19 death was defined as any record of death on the national Death Certificate Information System (SICO) with COVID-19 as the primary cause of death (ICD-10 *code U.071*) according to the WHO classification. SICO platform allows the issuance of a death certificate for each person who dies in Portugal [3]. A COVID-19 death was defined as any record of death on the national Death Certificate Information System (SICO) with COVID-19 as the primary cause of death (ICD-10 *code U.071*) according to the WHO classification. We followed the participants for 28 days after the sample collection day for the death outcome.

Ethical approval

The genomic surveillance of SARS-CoV-2 in Portugal is regulated by the Assistant Secretary of State and Health Executive Order (Despacho n.º 331/2021 of January 11, 2021). The research on genomic epidemiology of SARS-COV-2 received the clearance of the Ethics Committee of INSA on March 30, 2021.

Scotland

Data sources and linkage

In Scotland, health records are indexed by the 10-digit Community Health Index number (CHI), a unique patient identifier allocated uniquely to individuals receiving health care.

All cases who test positive for COVID-19 the disease are reported to the ECOSS system at Public Health Scotland, recording baseline characteristics such as age, sex, ethnicity, region of residence, and index of multiple deprivation (an area-level classification of relative deprivation). Data on all cases with positive test dates between 01 May 2021 and 31 March 2021, with a Scottish postcode, were extracted (n = 1,167,065 cases), and individually linked to variant classification results based on whole genome sequencing, provisional genotyping, or assessment of S gene positivity. The majority of exclusions were a result of the variant not being typed, with n = 225,658 post linkage and initial exclusions. After linkage to SARS-CoV-2 vaccination status, and further exclusions for incomplete or inconsistent vaccination status, n = 215,586 cases.

147,872 cases with date of test between 10 October 2021 and 14 February 2022, the time period during which both Delta and Omicron typed cases were observed, were included in the analysis.

Testing practices

During the inclusion period, community testing was widely available, including cost-free lateral flow testing of asymptomatic individuals in the community, cost-free PCR-based community testing of symptomatic individuals, and targeted routine testing of healthcare staff, hospitalised individuals and international travellers.

Variant classification

Approximately 10% of all positive COVID-19 tests were sequenced across Scotland during the study period. The variants of confirmed specimens were classified based on whole genome sequencing, allele specific PCR testing, or S gene positivity. Specimens were selected for whole genome

sequencing or allele specific PCR testing based on geographically weighted population-level sampling of community cases, and targeted selection of recent international travellers, fully vaccinated cases, immunocompromised patients, and hospitalised patients. This variant classification was supplemented with S gene positivity data for specimens from Scotland's community testing programme, which was available for approximately 90% of PCR positive results.

The Delta variant is associated with S gene positivity and the Omicron BA.1 variant with S gene negativity, but S gene status is not specific to these two variants. Therefore, the inclusion period for the analysis was chosen to ensure a sufficient prevalence of both variants to ensure high predictive values when S gene positivity/negativity was used to call variants as Delta or Omicron.

Outcome data and definitions

Hospital admissions were identified from the General Acute Inpatient and Day Case - Scottish Morbidity Record (SMR01). This is a fully validated hospital admissions dataset created after a patient has been discharged from hospital and contains CHI, patient-level demographic details (treatment location, age, gender *etc*), completed ICD-10 codes and several other variables related to a patient's stay in hospital. There is a time lag of around 3 months before full completion and availability. Patients with long hospital stays may not be included, as both an admission and discharge date are required to create a record.

A relevant hospital admission was defined as a hospital admission within 14 days of a positive COVID-19 test. A COVID-19 related hospital admission was defined as a hospital admission within 14 days of a positive COVID-19 test with U07.1 (laboratory confirmed COVID-19) or U07.2 (clinical suspicion of COVID-19) in the primary diagnosis field.

ICU admissions were identified from the Scottish Intensive Care Society Audit Group (SICSAG) dataset that documents all adult Intensive Care Unit (ICU) and Higher Dependency Unit (HDU) admissions in Scotland. A relevant ICU admission was defined as an ICU admission within 14 days of a positive COVID-19 test. A COVID-19 related ICU admission was defined as a patient aged over 16 years who had been admitted to ICU within 14 days of a positive COVID-19 test, if U07.1 or U07.2 was entered as the patient's primary diagnosis for admission.

Data on deaths were obtained from National Records Scotland (NRS), an electronic record of all deaths registered within Scotland. This contains data on cause, time and place of death. Any cause mortality was defined as any death within 28 days of a positive COVID-19 test. COVID-19 specific mortality was defined as any death within 28 days of a positive COVID-19 test with U07.1 or U07.2 included within their cause of death codes (at any position).

Ethical approval

This surveillance analysis was carried out by Public Health Scotland as part of its responsibility for the surveillance of respiratory pathogens in the population, and in accordance with UK GDPR Article 9 (2) i – the processing of personal data for the performance of a task for reasons of public interest in the area of public health.

Supplementary Figure S1. Inclusion summary flowchart for Denmark.

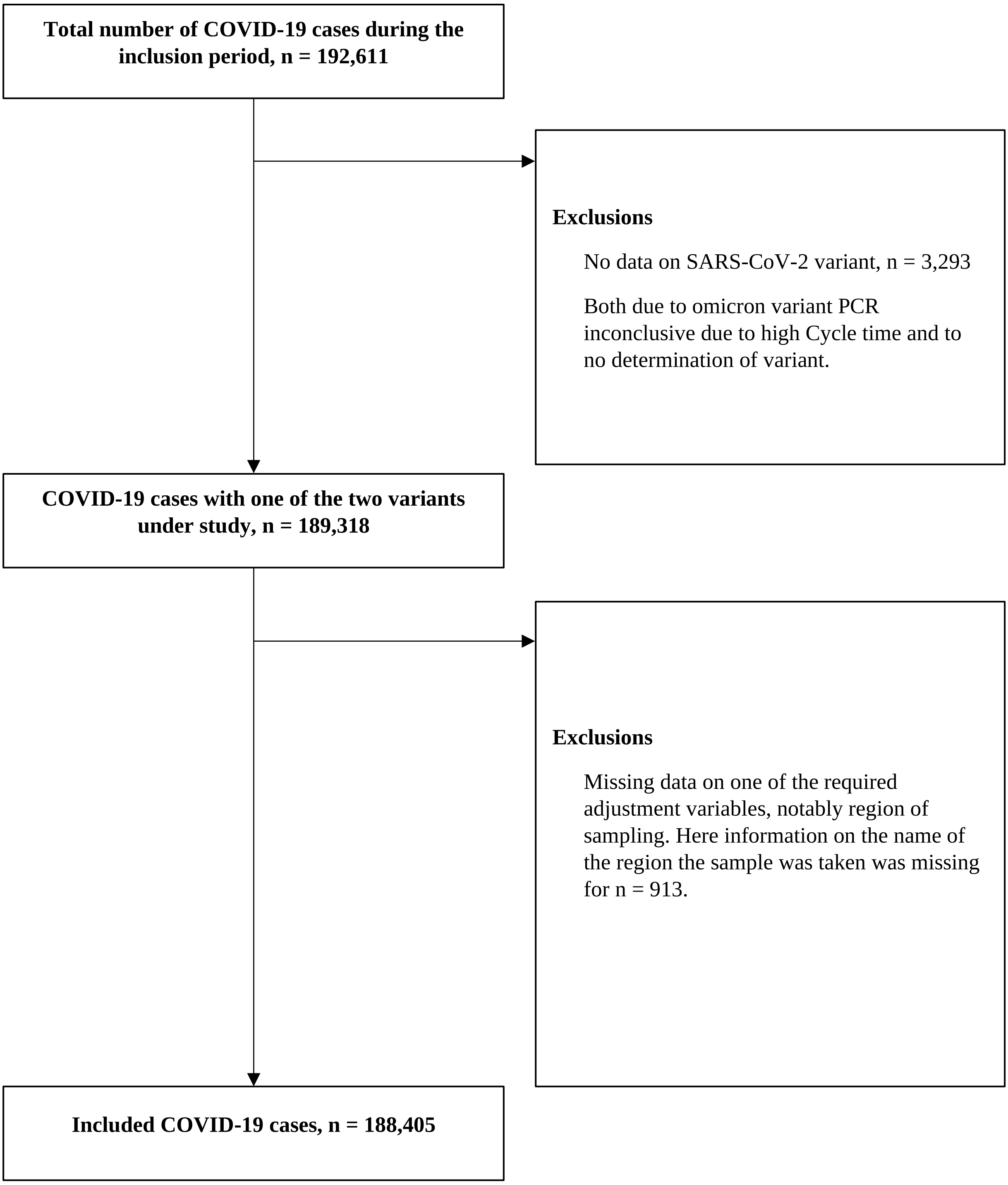

Supplementary Figure S2. Inclusion summary flowchart for England.

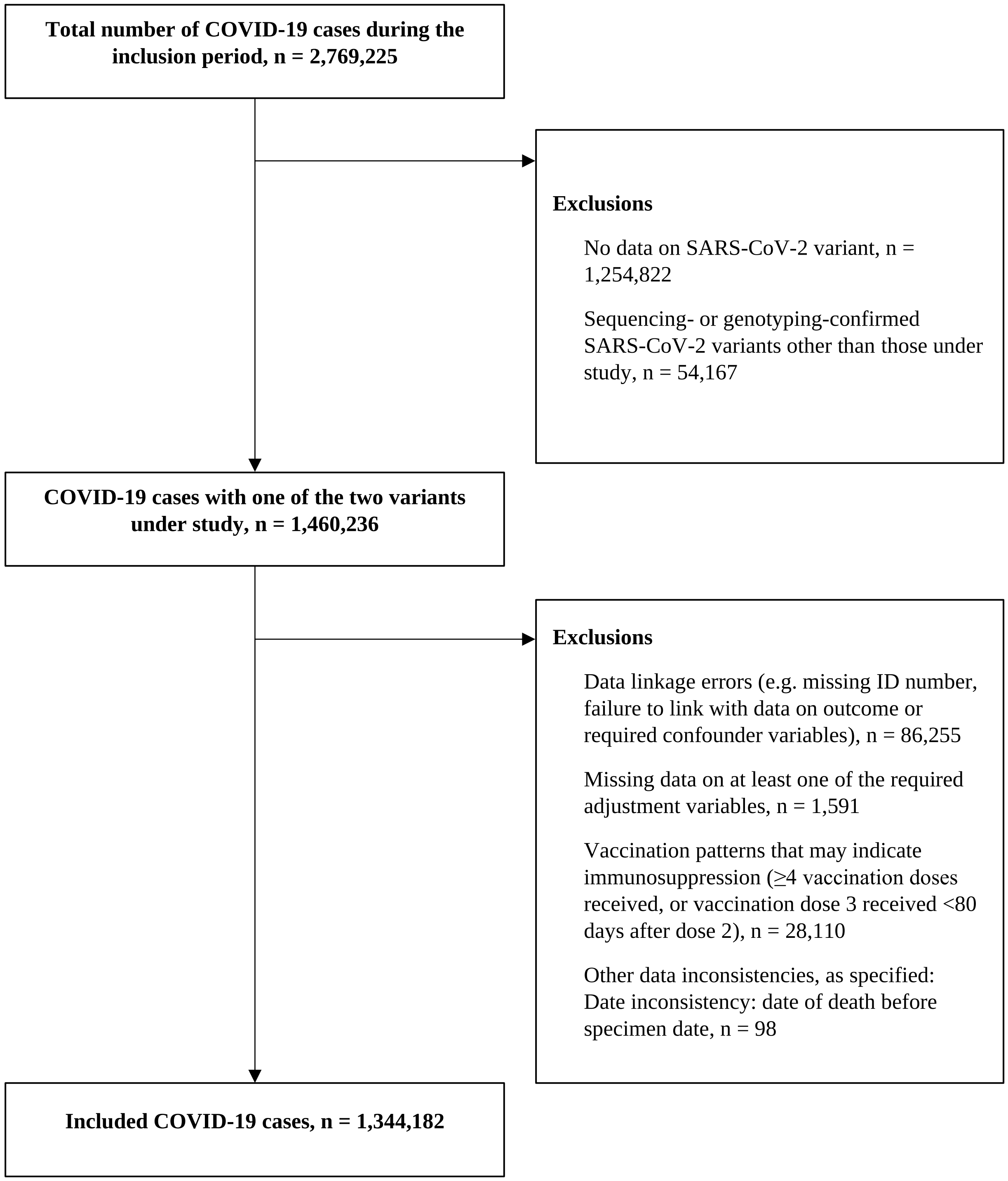

Supplementary Figure S3. Inclusion summary flowchart for Luxembourg.

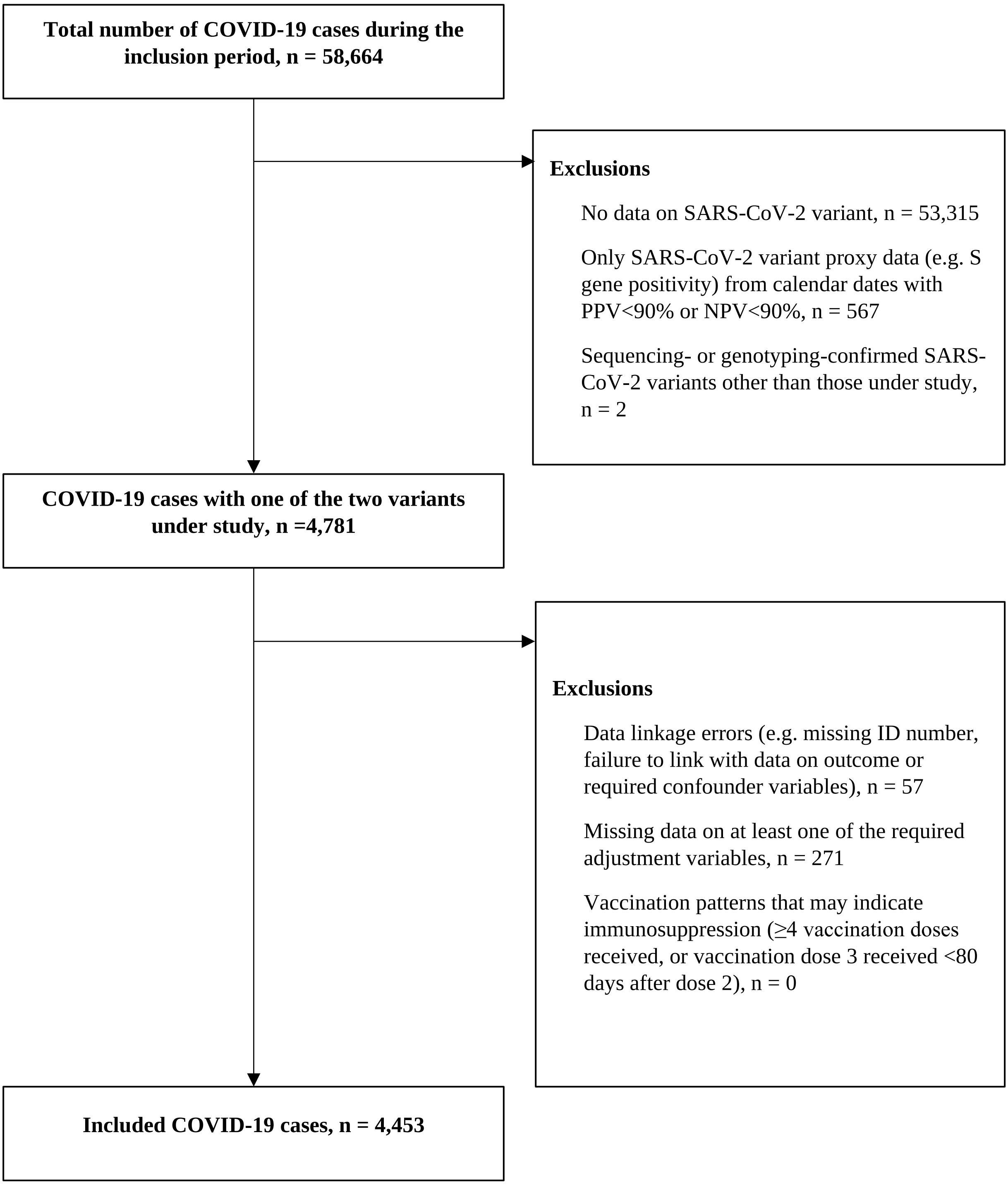

Supplementary Figure S4. Inclusion summary flowchart for Norway.

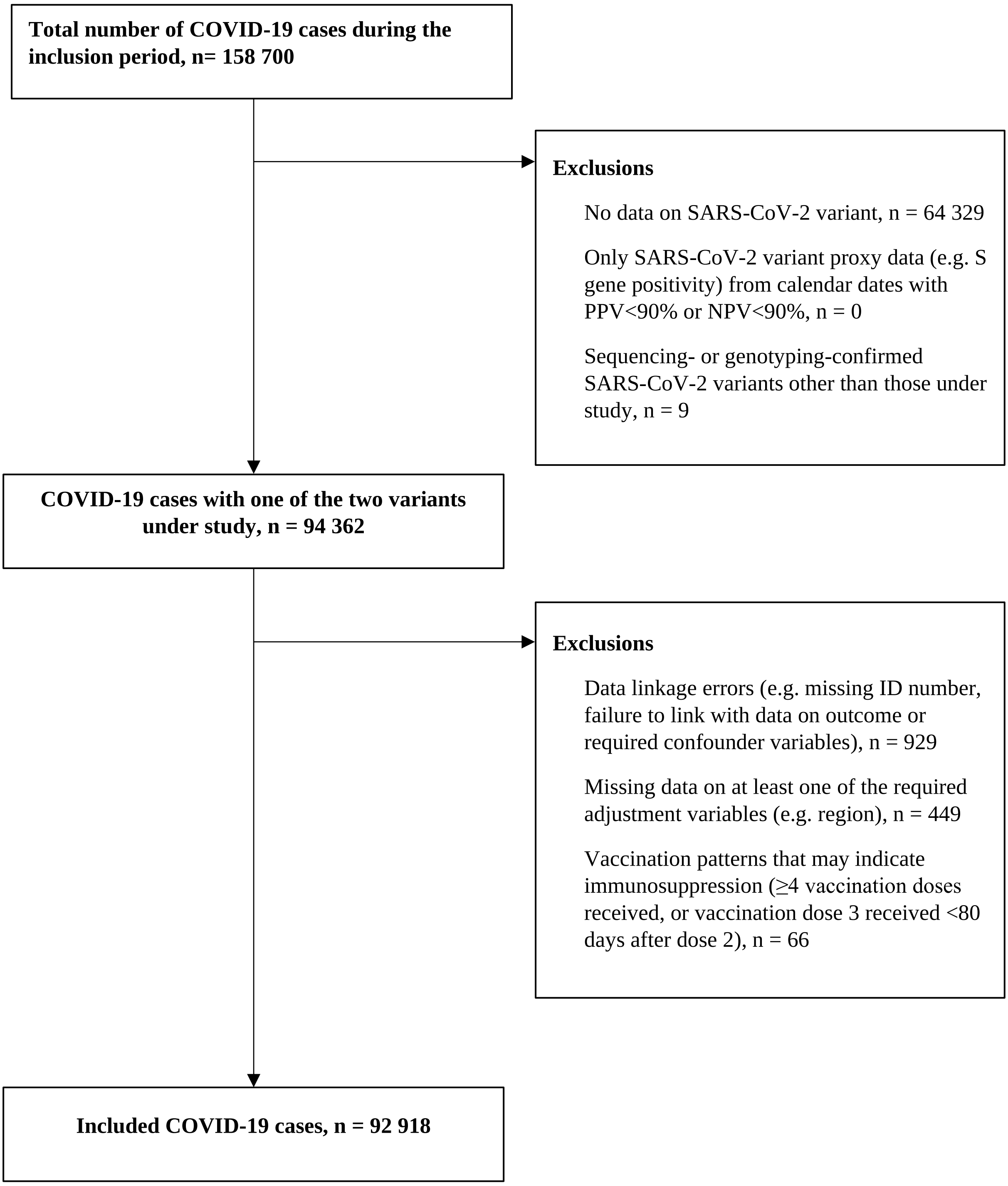

Supplementary Figure S5. Inclusion summary flowchart for Portugal.

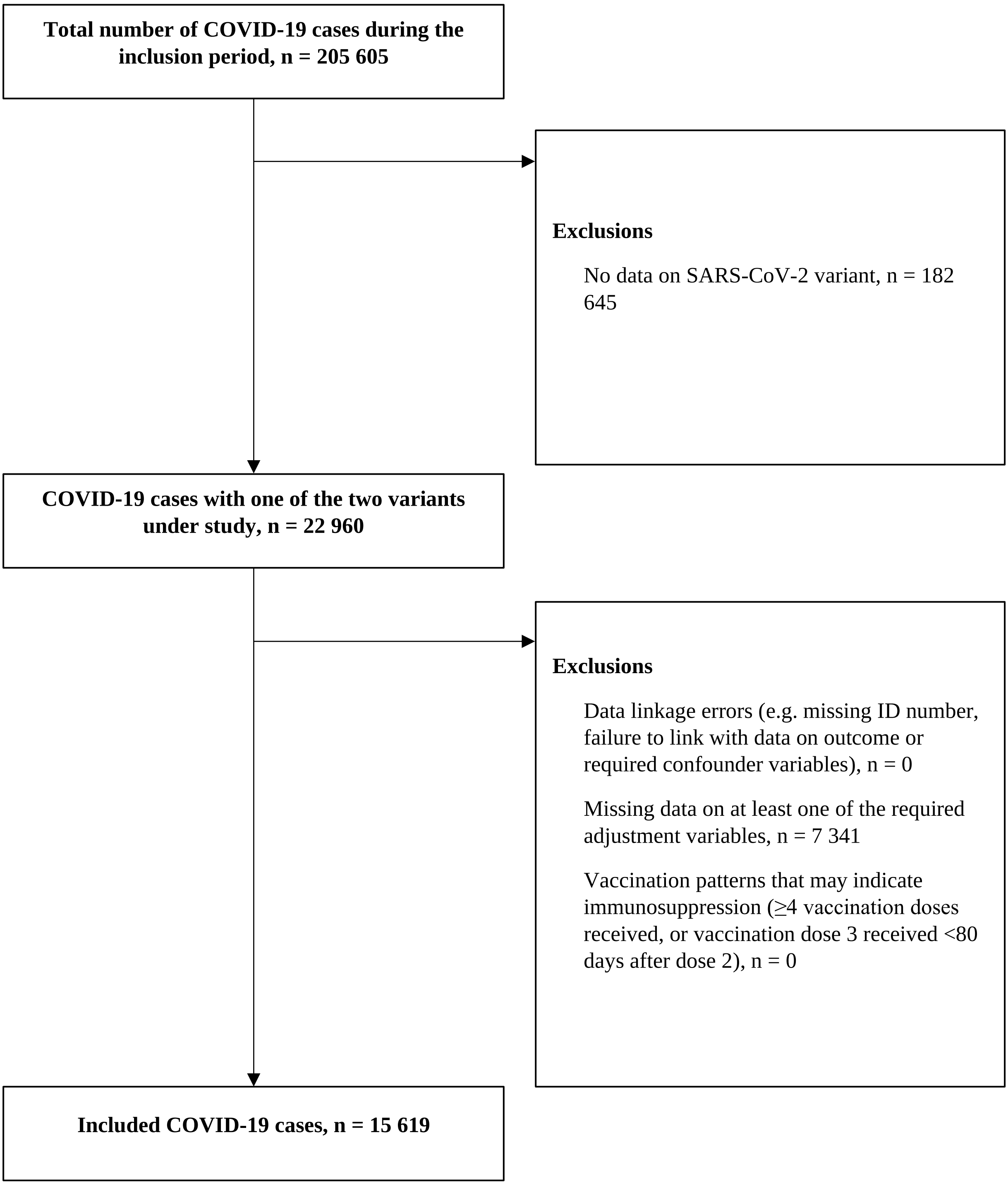

Supplementary Figure S6. Inclusion summary flowchart for Scotland.

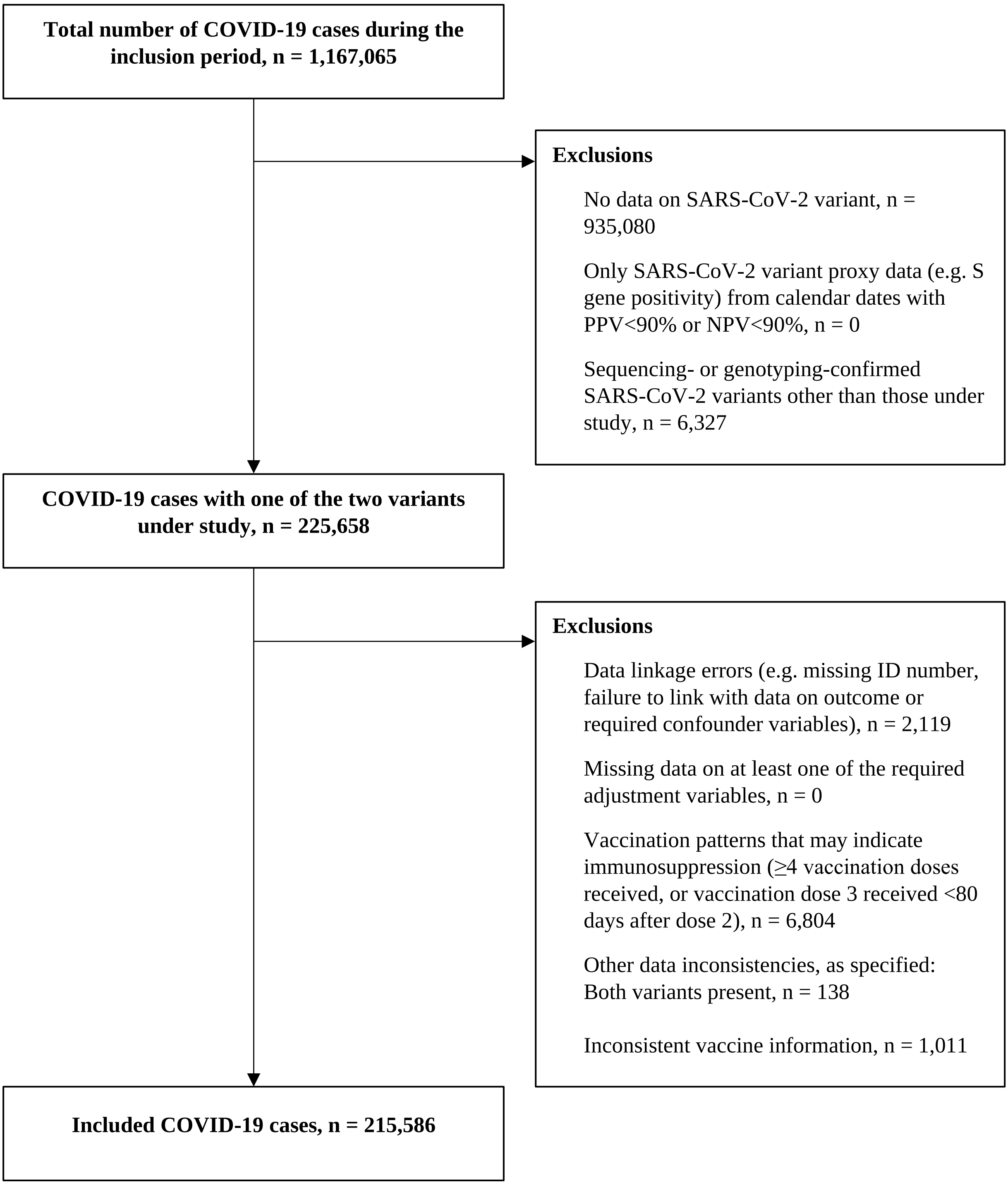

Supplementary Table S1. Summary of available outcomes and adjustment variables, by country.

Category	Variable	Country					
		**Denmark**	**England**	**Luxembourg**	**Norway**	**Portugal**	**Scotland**
**Outcome**	Hospital admission within 14 days after positive test	Available: Hospital admission due to COVID-19 (according to recorded ICD-10 codes) reported through three data sources, including hospital visits lasting longer than 12 hours, or lasting shorter than 12 hours but where a bed was assigned to the patient.	Available: Any hospital admission as recorded in two data sources covering admissions via emergency care or any hospital department where the time between admission and discharge was ≥1 day.	Available: Hospital admission for reasons potentially related to COVID-19 as determined and reported by hospitals.	Available: Hospital admission due to COVID-19 as reported to the national pandemic registry to which reporting was mandatory for hospitals during the study period, including cases who tested positive before admission or cases who tested positive during an ongoing hospitalisation.	Available: Any hospital admission as reported to a central hospital database.	Available: Hospital admissions due to COVID-19 (according to recorded ICD-10 codes).
	ICU admission within 14 days after positive test	Available: Hospital admission as defined above, to an ICU ward (as determined by hospital department coding).	--	Available: Hospital admission as defined above, to an ICU ward.	Available: Admission to an ICU ward as recorded in the above pandemic registry, which fulfilled one of five criteria: (1) length of stay >24 hours in intensive care, (2) required mechanical ventilation, (3) transferred between intensive care wards, (4) persistent administration of vasoactive medication, or (5) length of stay under 24 hours but passed away during stay in intensive care.	Available: ICU admission as reported to the above central hospital database.	Available: Recorded ICU admission during hospital stay due to COVID-19 (as defined above).

	Death within 28 days after positive test	Available:<br>Death as recorded in the population Civil Registration System and the Cause of Death Register.	Available:<br>Deaths collected through hospital report and linkage with health records and death registrations.	Available: Death as recorded in population register with COVID-19 as primary cause of death.	Available:<br>Death as recorded in population register, with COVID-19 as primary or contributing cause of death on the death certificate.	Available:<br>Death as recorded in population register, with COVID-19 as primary cause of death on the death certificate.	Available:<br>Death as recorded in population register, with COVID-19 as primary or contributing cause of death on the death certificate.
**Required adjustment variable**	Sex	Available with protocol-requested categories	Available with protocol-requested categories	Available with protocol-requested categories	Available with protocol-requested categories	Available with protocol-requested categories	Available with protocol-requested categories
	Age at diagnosis	Available (exact age)	Available (exact age)	Available (exact age)	Available (exact age)	Available (exact age)	Available (exact age)
	Calendar date of positive test	Available (exact date)	Available (exact date)	Available (exact date)	Available (exact date)	Available (exact date)	Available (exact date)
	Area of residence	Available:<br>Copenhagen, Midtjylland, Nordjylland, Sjælland, Syddanmark	Available:<br>East of England, London, Midlands, North East and Yorkshire, North West, South East, South West	Available:<br>Luxembourg (whole country)	Available:<br>East Norway, Oslo-wide area, South and West Norway, Mid-Norway, North Norway	Available:<br>Algarve & Alentejo, Centro, Lisboa e Vale do Tejo, Norte	Available:<br>Scotland (whole country)
	Vaccination status	Available with protocol-requested categories	Available with protocol-requested categories	Available with protocol-requested categories	Available with protocol-requested categories	Available with protocol-requested categories	Available with protocol-requested categories
**Highly desired adjustment variable**	Reinfection status	Available with protocol-requested categories	Available with protocol-requested categories	Available with protocol-requested categories	Available with protocol-requested categories	Available with protocol-requested categories	Available with protocol-requested categories
**Desired adjustment variable**	Ethnicity/country of birth	Available:<br>Danish, second-generation immigrant, first-generation immigrant	Available:<br>White, Asian, Black, Mixed/Other, Unknown	--	Available:<br>Born in Norway, Not born in Norway, Unknown	Available:<br>Portuguese, Other	Available:<br>White, Asian, Black, Mixed/Multiple, Other, Unknown
	Socioeconomic status/deprivation indicator	--	Available:<br>Index of multiple deprivation decile (10 categories)	--	Available:<br>Not overcrowded home, Overcrowded home, Overcrowding status unknown	--	Available:<br>Index of multiple deprivation quintile (5 categories)

Comorbidity	Available: Number of comorbidities in the past 5 years (0 vs ≥1). The comorbidities include diabetes, adiposity, cancer, neurological diseases, nephrological diseases, haematological diseases, cardiac disease, respiratory diseases, immunological diseases, and other comorbid diseases.	--	--	Available: No underlying comorbidities, Medium-risk comorbidity, High-risk comorbidity [4]	--	--
International travel within 14 days before positive test	--	--	Available: No international travel within 14 days, International travel within 14 days	--	--	--

Supplementary Table S2. Descriptive outcome frequencies for included COVID-19 Delta or Omicron cases, by country

Country	Outcome	n/N (%)	
		**Delta**	**Omicron**
Denmark	Hospital admission (COVID-19-specific)	1656/149689 (1.1%)	143/38716 (0.37%)
	Hospital admission (any cause)	2344/149689 (1.6%)	258/38716 (0.67%)
	ICU admission (any cause)	185/149689 (0.12%)	6/38716 (0.015%)
	Death (any cause)	264/149689 (0.18%)	13/38716 (0.034%)
England	Hospital admission (any cause)	8444/429231 (2.0%)	8364/914951 (0.91%)
	Death (any cause)	1342/429231 (0.31%)	990/914951 (0.11%)
Luxembourg	Hospital admission (COVID-19-specific)	70/1286 (5.4%)	86/3167 (2.7%)
	ICU admission (COVID-19-specific)	10/1286 (0.78%)	4/3167 (0.13%)
	Death (COVID-19-specific)	10/1286 (0.78%)	14/3167 (0.44%)
Norway	Hospital admission (COVID-19-specific)	520/52091 (1.0%)	90/40827 (0.22%)
	ICU admission (COVID-19-specific)	151/52091 (0.29%)	10/40827 (0.024%)
	Death (COVID-19-specific)	105/52091 (0.20%)	17/40827 (0.042%)
Portugal	Hospital admission (any cause)	139/9150 (1.5%)	15/6469 (0.23%)
	Death (any cause)	24/9150 (0.26%)	0/6469 (0%)
Scotland	Hospital admission (COVID-19-specific)	406/35534 (1.1%)	117/47744 (0.25%)
	Hospital admission (any cause)	510/35534 (1.4%)	265/47744 (0.56%)
	ICU admission (COVID-19-specific)	106/35534 (0.30%)	9/47744 (0.019%)
	ICU admission (any cause)	116/35534 (0.33%)	17/47744 (0.036%)
	Death (COVID-19-specific)	70/35534 (0.20%)	9/47744 (0.019%)
	Death (any cause)	72/35534 (0.20%)	11/47744 (0.023%)

Supplementary Table S3. Descriptive characteristics of included Delta or Omicron cases, by country

Country	Category	Variable	Delta	Omicron	Overall
Denmark			(N=149689)	(N=38716)	(N=188405)
	Required adjustment variable	Sex			
		Female	75363 (50.3%)	19450 (50.2%)	94813 (50.3%)
		Male	74326 (49.7%)	19266 (49.8%)	93592 (49.7%)
		Age (years)			
		Mean (SD)	32.9 (20.9)	33.4 (16.7)	33.0 (20.1)
		Median (Q1-Q3)	32.0 (12.0-49.0)	29.0 (21.0-46.0)	31.0 (14.0-48.0)
		0-19	53530 (35.8%)	8915 (23.0%)	62445 (33.1%)
		20-39	38700 (25.9%)	16980 (43.9%)	55680 (29.6%)
		40-49	23584 (15.8%)	5519 (14.3%)	29103 (15.4%)
		50-59	17075 (11.4%)	4706 (12.2%)	21781 (11.6%)
		60-69	9895 (6.6%)	1846 (4.8%)	11741 (6.2%)
		≥70	6905 (4.6%)	750 (1.9%)	7655 (4.1%)
		Calendar week			
		ISO week 46, 2021	<3668 (2.4%)	<5 (0.0%)	3668 (1.9%)
		ISO week 47, 2021	<28750 (19.2%)	<25 (0.1%)	28766 (15.3%)
		ISO week 48, 2021	33289 (22.2%)	521 (1.3%)	33810 (17.9%)
		ISO week 49, 2021	41842 (28.0%)	5951 (15.4%)	47793 (25.4%)
		ISO week 50, 2021	42147 (28.2%)	32221 (83.2%)	74368 (39.5%)
		Area of residence			
		Hovedstaden	65926 (44.0%)	23573 (60.9%)	89499 (47.5%)
		Midtjylland	25277 (16.9%)	6790 (17.5%)	32067 (17.0%)
		Nordjylland	10603 (7.1%)	1816 (4.7%)	12419 (6.6%)
		Sjælland	23177 (15.5%)	3398 (8.8%)	26575 (14.1%)
		Syddanmark	24706 (16.5%)	3139 (8.1%)	27845 (14.8%)
		Vaccination status at the time of positive test			
		Unvaccinated	60964 (40.7%)	5308 (13.7%)	66272 (35.2%)
		≥0d after first dose	8209 (5.5%)	1727 (4.5%)	9936 (5.3%)
		≥14d after second dose	49034 (32.8%)	20407 (52.7%)	69441 (36.9%)
		≥153d after second dose	28151 (18.8%)	9576 (24.7%)	37727 (20.0%)
		≥14d after third dose	3331 (2.2%)	1698 (4.4%)	5029 (2.7%)
	Highly desired adjustment variable	Reinfection status			
		First infection	147828 (98.8%)	36570 (94.5%)	184398 (97.9%)
		Reinfection	1861 (1.2%)	2146 (5.5%)	4007 (2.1%)
	Desired adjustment variable	Ethnicity/Country of birth			
		Danish	122052 (81.5%)	33176 (85.7%)	155228 (82.4%)
		Second-generation immigrant	8609 (5.8%)	1299 (3.4%)	9908 (5.3%)
		First-generation immigrant	19028 (12.7%)	4241 (11.0%)	23269 (12.4%)
		Comorbidity			
		No comorbidities	125556 (83.9%)	33765 (87.2%)	159321 (84.6%)

		≥1 comorbidity	24133 (16.1%)	4951 (12.8%)	29084 (15.4%)
England			(N=429231)	(N=914951)	(N=1344182)
	Required adjustment variable	Sex			
		Female	223332 (52.0%)	490812 (53.6%)	714144 (53.1%)
		Male	205899 (48.0%)	424139 (46.4%)	630038 (46.9%)
		Age (years)			
		Mean (SD)	30.5 (18.5)	35.3 (15.9)	33.7 (16.9)
		Median (Q1-Q3)	31.0 (12.0-45.0)	33.0 (24.0-46.0)	33.0 (21.0-46.0)
		0-19	155186 (36.2%)	133918 (14.6%)	289104 (21.5%)
		20-39	122957 (28.6%)	448159 (49.0%)	571116 (42.5%)
		40-49	79133 (18.4%)	147319 (16.1%)	226452 (16.8%)
		50-59	47845 (11.1%)	113381 (12.4%)	161226 (12.0%)
		60-69	17597 (4.1%)	52007 (5.7%)	69604 (5.2%)
		≥70	6513 (1.5%)	20167 (2.2%)	26680 (2.0%)
		Calendar week			
		ISO week 48, 2021	157378 (36.7%)	3459 (0.4%)	160837 (12.0%)
		ISO week 49, 2021	139431 (32.5%)	34250 (3.7%)	173681 (12.9%)
		ISO week 50, 2021	81215 (18.9%)	198634 (21.7%)	279849 (20.8%)
		ISO week 51, 2021	38749 (9.0%)	361719 (39.5%)	400468 (29.8%)
		ISO week 52, 2021	12458 (2.9%)	316889 (34.6%)	329347 (24.5%)
		Area of residence			
		East of England	48108 (11.2%)	81063 (8.9%)	129171 (9.6%)
		London	47528 (11.1%)	186165 (20.3%)	233693 (17.4%)
		Midlands	77648 (18.1%)	143163 (15.6%)	220811 (16.4%)
		North East and Yorkshire	79609 (18.5%)	160866 (17.6%)	240475 (17.9%)
		North West	58973 (13.7%)	170838 (18.7%)	229811 (17.1%)
		South East	72046 (16.8%)	119925 (13.1%)	191971 (14.3%)
		South West	45319 (10.6%)	52931 (5.8%)	98250 (7.3%)
		Vaccination status at the time of positive test			
		Unvaccinated	179523 (41.8%)	161170 (17.6%)	340693 (25.3%)
		≥0d after first dose	34028 (7.9%)	60682 (6.6%)	94710 (7.0%)
		≥14d after second dose	60614 (14.1%)	230801 (25.2%)	291415 (21.7%)
		≥153d after second dose	137944 (32.1%)	281181 (30.7%)	419125 (31.2%)
		≥14d after third dose	17122 (4.0%)	181117 (19.8%)	198239 (14.7%)
	Highly desired adjustment variable	Reinfection status			
		First infection	423850 (98.7%)	830188 (90.7%)	1254038 (93.3%)
		Reinfection	5381 (1.3%)	84763 (9.3%)	90144 (6.7%)
	Desired adjustment variable	Ethnicity/Country of birth			
		White	363157 (84.6%)	733624 (80.2%)	1096781 (81.6%)
		Asian	26279 (6.1%)	66974 (7.3%)	93253 (6.9%)
		Black	8592 (2.0%)	42911 (4.7%)	51503 (3.8%)
		Mixed/Other	15217 (3.5%)	35850 (3.9%)	51067 (3.8%)
		Unknown	15986 (3.7%)	35592 (3.9%)	51578 (3.8%)

		Socioeconomic status/deprivation indicator			
		IMD decile 1	41076 (9.6%)	82873 (9.1%)	123949 (9.2%)
		IMD decile 2	38467 (9.0%)	92257 (10.1%)	130724 (9.7%)
		IMD decile 3	39691 (9.2%)	98100 (10.7%)	137791 (10.3%)
		IMD decile 4	41442 (9.7%)	93182 (10.2%)	134624 (10.0%)
		IMD decile 5	41687 (9.7%)	91302 (10.0%)	132989 (9.9%)
		IMD decile 6	43922 (10.2%)	91130 (10.0%)	135052 (10.0%)
		IMD decile 7	43863 (10.2%)	90912 (9.9%)	134775 (10.0%)
		IMD decile 8	45208 (10.5%)	93050 (10.2%)	138258 (10.3%)
		IMD decile 9	46155 (10.8%)	92217 (10.1%)	138372 (10.3%)
		IMD decile 10	47720 (11.1%)	89928 (9.8%)	137648 (10.2%)
Luxembourg			(N=1286)	(N=3167)	(N=4453)
	Required adjustment variable	Sex			
		Female	639 (49.7%)	1634 (51.6%)	2273 (51.0%)
		Male	647 (50.3%)	1533 (48.4%)	2180 (49.0%)
		Age (years)			
		Mean (SD)	36.1 (21.1)	32.9 (20.5)	33.8 (20.7)
		Median (Q1-Q3)	37.0 (19.0-51.0)	32.0 (16.0-45.0)	33.0 (17.0-47.0)
		0-19	326 (25.4%)	918 (29.0%)	1244 (27.9%)
		20-39	389 (30.2%)	1118 (35.3%)	1507 (33.8%)
		40-49	222 (17.3%)	536 (16.9%)	758 (17.0%)
		50-59	189 (14.7%)	307 (9.7%)	496 (11.1%)
		60-69	81 (6.3%)	115 (3.6%)	196 (4.4%)
		≥70	79 (6.1%)	173 (5.5%)	252 (5.7%)
		Calendar week			
		ISO week 49, 2021	404 (31.4%)	5 (0.2%)	409 (9.2%)
		ISO week 50, 2021	482 (37.5%)	66 (2.1%)	548 (12.3%)
		ISO week 51, 2021	240 (18.7%)	255 (8.1%)	495 (11.1%)
		ISO week 52, 2021	87 (6.8%)	503 (15.9%)	590 (13.2%)
		ISO week 1, 2022	50 (3.9%)	682 (21.5%)	732 (16.4%)
		ISO week 2, 2022	17 (1.3%)	668 (21.1%)	685 (15.4%)
		ISO week 3, 2022	6 (0.5%)	988 (31.2%)	994 (22.3%)
		Area of residence			
		Luxembourg	1286 (100%)	3167 (100%)	4453 (100%)
		Vaccination status at the time of positive test			
		Unvaccinated	683 (53.1%)	1072 (33.8%)	1755 (39.4%)
		≥0d after first dose	83 (6.5%)	271 (8.6%)	354 (7.9%)
		≥14d after second dose	146 (11.4%)	458 (14.5%)	604 (13.6%)
		≥153d after second dose	352 (27.4%)	948 (29.9%)	1300 (29.2%)
		≥14d after third dose	22 (1.7%)	418 (13.2%)	440 (9.9%)
	Highly desired adjustment variable	Reinfection status			
		First infection	1253 (97.4%)	2867 (90.5%)	4120 (92.5%)
		Reinfection	33 (2.6%)	300 (9.5%)	333 (7.5%)

	Desired adjustment variable	International travel within 14 days before positive test			
		Yes	50 (3.9%)	175 (5.5%)	225 (5.1%)
		No	1236 (96.1%)	2992 (94.5%)	4228 (94.9%)
Norway			(N=52091)	(N=40827)	(N=92918)
	Required adjustment variable	Sex			
		Female	25865 (49.7%)	20297 (49.7%)	46162 (49.7%)
		Male	26226 (50.3%)	20530 (50.3%)	46756 (50.3%)
		Age (years)			
		Mean (SD)	29.9 (19.5)	31.4 (17.0)	30.5 (18.5)
		Median (Q1-Q3)	30.0 (11.0-44.0)	29.0 (19.0-43.0)	29.0 (14.0-44.0)
		0-19	20413 (39.2%)	10413 (25.5%)	30826 (33.2%)
		20-39	14221 (27.3%)	17914 (43.9%)	32135 (34.6%)
		40-49	8664 (16.6%)	5811 (14.2%)	14475 (15.6%)
		50-59	4864 (9.3%)	4303 (10.5%)	9167 (9.9%)
		60-69	2597 (5.0%)	1566 (3.8%)	4163 (4.5%)
		≥70	1332 (2.6%)	820 (2.0%)	2152 (2.3%)
		Calendar week			
		ISO week 49, 2021	19964 (38.3%)	1276 (3.1%)	21240 (22.9%)
		ISO week 50, 2021	17016 (32.7%)	3262 (8.0%)	20278 (21.8%)
		ISO week 51, 2021	8033 (15.4%)	5045 (12.4%)	13078 (14.1%)
		ISO week 52, 2021	4731 (9.1%)	11612 (28.4%)	16343 (17.6%)
		ISO week 1, 2022	2347 (4.5%)	19632 (48.1%)	21979 (23.7%)
		Area of residence			
		East Norway	11878 (22.8%)	6483 (15.9%)	18361 (19.8%)
		Oslo-wide area	22998 (44.1%)	23816 (58.3%)	46814 (50.4%)
		South and West Norway	12906 (24.8%)	7659 (18.8%)	20565 (22.1%)
		Mid-Norway	3583 (6.9%)	2262 (5.5%)	5845 (6.3%)
		North Norway	726 (1.4%)	607 (1.5%)	1333 (1.4%)
		Vaccination status at the time of positive test			
		Unvaccinated	23188 (44.5%)	8421 (20.6%)	31609 (34.0%)
		≥0d after first dose	4602 (8.8%)	3956 (9.7%)	8558 (9.2%)
		≥14d after second dose	16346 (31.4%)	20442 (50.1%)	36788 (39.6%)
		≥153d after second dose	7124 (13.7%)	5294 (13.0%)	12418 (13.4%)
		≥14d after third dose	831 (1.6%)	2714 (6.6%)	3545 (3.8%)
	Highly desired adjustment variable	Reinfection status			
		First infection	51889 (99.6%)	39127 (95.8%)	91016 (98.0%)
		Reinfection	202 (0.4%)	1700 (4.2%)	1902 (2.0%)
	Desired adjustment variable	Ethnicity/Country of birth			
		Born in Norway	38992 (74.9%)	28808 (70.6%)	67800 (73.0%)
		Not born in Norway	12367 (23.7%)	11654 (28.5%)	24021 (25.9%)
		Unknown	732 (1.4%)	365 (0.9%)	1097 (1.2%)

		Socioeconomic status/deprivation indicator			
		Not overcrowded home	39147 (75.2%)	30113 (73.8%)	69260 (74.5%)
		Overcrowded home	9186 (17.6%)	7453 (18.3%)	16639 (17.9%)
		Overcrowding status unknown	3758 (7.2%)	3261 (8.0%)	7019 (7.6%)
		Comorbidity			
		No underlying comorbidities	45994 (88.3%)	36744 (90.0%)	82738 (89.0%)
		Medium-risk comorbidity	5547 (10.6%)	3740 (9.2%)	9287 (10.0%)
		High-risk comorbidity	550 (1.1%)	343 (0.8%)	893 (1.0%)
Portugal			(N=9150)	(N=6469)	(N=15619)
	Required adjustment variable	Sex			
		Female	4505 (49.2%)	3310 (51.2%)	7815 (50.0%)
		Male	4645 (50.8%)	3159 (48.8%)	7804 (50.0%)
		Age (years)			
		Mean (SD)	43.3 (15.8)	37.1 (14.8)	40.7 (15.7)
		Median (Q1-Q3)	42.1 (30.7-54.0)	34.4 (24.4-47.4)	39.6 (27.5-51.2)
		0-19	403 (4.4%)	630 (9.7%)	1033 (6.6%)
		20-39	3692 (40.3%)	3250 (50.2%)	6942 (44.4%)
		40-49	2100 (23.0%)	1295 (20.0%)	3395 (21.7%)
		50-59	1492 (16.3%)	801 (12.4%)	2293 (14.7%)
		60-69	945 (10.3%)	336 (5.2%)	1281 (8.2%)
		≥70	518 (5.7%)	157 (2.4%)	675 (4.3%)
		Calendar week			
		ISO week 48, 2021	296 (3.2%)	13 (0.2%)	309 (2.0%)
		ISO week 49, 2021	3586 (39.2%)	209 (3.2%)	3795 (24.3%)
		ISO week 50, 2021	3104 (33.9%)	1129 (17.5%)	4233 (27.1%)
		ISO week 51, 2021	1942 (21.2%)	4142 (64.0%)	6084 (39.0%)
		ISO week 52, 2021	222 (2.4%)	976 (15.1%)	1198 (7.7%)
		Area of residence			
		Algarve or Alentejo	567 (6.2%)	243 (3.8%)	810 (5.2%)
		Centro	1011 (11.0%)	372 (5.8%)	1383 (8.9%)
		Lisboa e Vale do Tejo	1778 (19.4%)	2298 (35.5%)	4076 (26.1%)
		Norte	5794 (63.3%)	3556 (55.0%)	9350 (59.9%)
		Vaccination status at the time of positive test			
		Unvaccinated	972 (10.6%)	751 (11.6%)	1723 (11.0%)
		≥0d after first dose	1821 (19.9%)	1208 (18.7%)	3029 (19.4%)
		≥14d after second dose	3288 (35.9%)	2533 (39.2%)	5821 (37.3%)
		≥153d after second dose	2855 (31.2%)	1683 (26.0%)	4538 (29.1%)
		≥14d after third dose	214 (2.3%)	294 (4.5%)	508 (3.3%)
	Highly desired adjustment variable	Reinfection status			
		First infection	9011 (98.5%)	6023 (93.1%)	15034 (96.3%)
		Reinfection	139 (1.5%)	446 (6.9%)	585 (3.7%)
	Desired adjustment variable	Ethnicity/Country of birth			

		Portugal	8879 (97.0%)	6289 (97.2%)	15168 (97.1%)
		Other	271 (3.0%)	180 (2.8%)	451 (2.9%)
Scotland			(N=35534)	(N=47744)	(N=83278)
	Required adjustment variable	Sex			
		Male	17217 (48.5%)	21442 (44.9%)	38659 (46.4%)
		Female	18317 (51.5%)	26302 (55.1%)	44619 (53.6%)
		Age (years)			
		Mean (SD)	33.3 (19.5)	33.2 (17.0)	33.3 (18.1)
		Median (Q1-Q3)	35.0 (13.0-49.0)	32.0 (21.0-45.0)	33.0 (18.0-47.0)
		0-19	11530 (32.4%)	10860 (22.7%)	22390 (26.9%)
		20-39	9116 (25.7%)	20211 (42.3%)	29327 (35.2%)
		40-49	6155 (17.3%)	7292 (15.3%)	13447 (16.1%)
		50-59	5311 (14.9%)	5731 (12.0%)	11042 (13.3%)
		60-69	2743 (7.7%)	2918 (6.1%)	5661 (6.8%)
		≥70	679 (1.9%)	732 (1.5%)	1411 (1.7%)
		Calendar week			
		ISO week 41, 2021	1778 (5.0%)	10 (0.0%)	1788 (2.1%)
		ISO week 42, 2021	2495 (7.0%)	16 (0.0%)	2511 (3.0%)
		ISO week 43, 2021	4407 (12.4%)	12 (0.0%)	4419 (5.3%)
		ISO week 44, 2021	4031 (11.3%)	15 (0.0%)	4046 (4.9%)
		ISO week 45, 2021	3578 (10.1%)	39 (0.1%)	3617 (4.3%)
		ISO week 46, 2021	3475 (9.8%)	42 (0.1%)	3517 (4.2%)
		ISO week 47, 2021	5514 (15.5%)	80 (0.2%)	5594 (6.7%)
		ISO week 48, 2021	3500 (9.8%)	234 (0.5%)	3734 (4.5%)
		ISO week 49, 2021	4163 (11.7%)	1221 (2.6%)	5384 (6.5%)
		ISO week 50, 2021	1662 (4.7%)	2484 (5.2%)	4146 (5.0%)
		ISO week 51, 2021	471 (1.3%)	2447 (5.1%)	2918 (3.5%)
		ISO week 52, 2021	302 (0.8%)	5927 (12.4%)	6229 (7.5%)
		ISO week 1, 2022	90 (0.3%)	4352 (9.1%)	4442 (5.3%)
		ISO week 2, 2022	37 (0.1%)	4025 (8.4%)	4062 (4.9%)
		ISO week 3, 2022	16 (0.0%)	5533 (11.6%)	5549 (6.7%)
		ISO week 4, 2022	4 (0.0%)	3361 (7.0%)	3365 (4.0%)
		ISO week 5, 2022	9 (0.0%)	6683 (14.0%)	6692 (8.0%)
		ISO week 6, 2022	2 (0.0%)	11263 (23.6%)	11265 (13.5%)
		Area of residence			
		Scotland	35534 (100%)	47744 (100%)	83278 (100%)
		Vaccination status at the time of positive test			
		Unvaccinated	12855 (36.2%)	11699 (24.5%)	24554 (29.5%)
		≥0d after first dose	2961 (8.3%)	3180 (6.7%)	6141 (7.4%)
		≥14d after second dose	9154 (25.8%)	6788 (14.2%)	15942 (19.1%)
		≥153d after second dose	9723 (27.4%)	8508 (17.8%)	18231 (21.9%)
		≥14d after third dose	841 (2.4%)	17569 (36.8%)	18410 (22.1%)
	Highly desired adjustment variable	Reinfection status			
		First infection	35278 (99.3%)	43713 (91.6%)	78991 (94.9%)

		Reinfection	256 (0.7%)	4031 (8.4%)	4287 (5.1%)
	Desired adjustment variable	Ethnicity/Country of birth			
		White	32775 (92.2%)	43215 (90.5%)	75990 (91.2%)
		Asian	744 (2.1%)	1635 (3.4%)	2379 (2.9%)
		Black	199 (0.6%)	438 (0.9%)	637 (0.8%)
		Mixed/Multiple ethnic group	348 (1.0%)	473 (1.0%)	821 (1.0%)
		Other ethnic group	108 (0.3%)	233 (0.5%)	341 (0.4%)
		Unknown	1360 (3.8%)	1750 (3.7%)	3110 (3.7%)
		Socioeconomic status/deprivation indicator			
		IMD quintile 1	6773 (19.1%)	10524 (22.0%)	17297 (20.8%)
		IMD quintile 2	6969 (19.6%)	9995 (20.9%)	16964 (20.4%)
		IMD quintile 3	6763 (19.0%)	8661 (18.1%)	15424 (18.5%)
		IMD quintile 4	7486 (21.1%)	9337 (19.6%)	16823 (20.2%)
		IMD quintile 5	7543 (21.2%)	9227 (19.3%)	16770 (20.1%)

Supplementary Figure S7. Unadjusted hazard ratios for COVID-19 cases infected with the Omicron versus Delta variants, of (A) hospital admission (COVID-19-specific, where available, or otherwise due to any cause), (B) ICU admission (COVID-19-specific), or (C) death (COVID-19-specific, where available, or otherwise due to any cause).

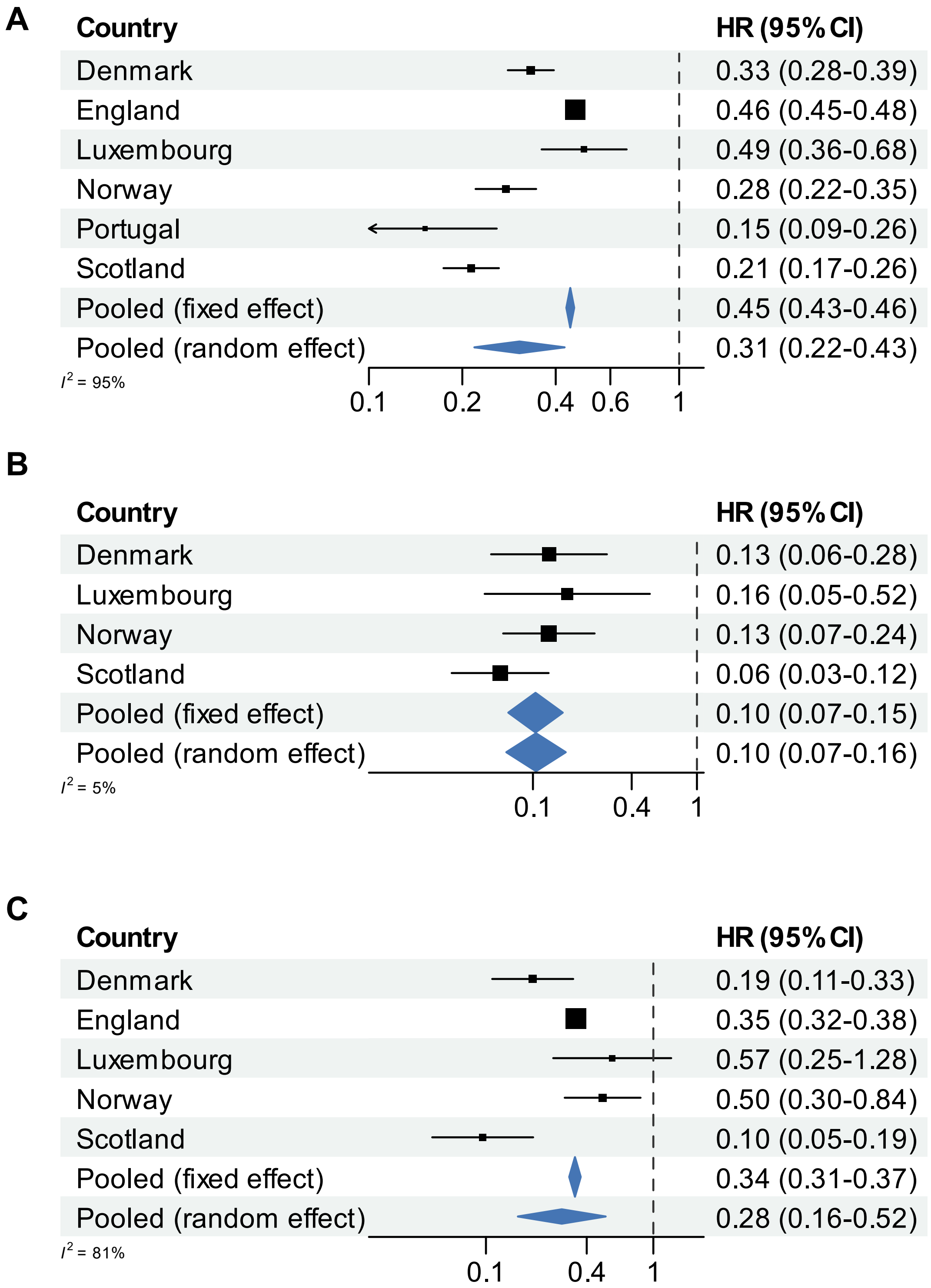

Supplementary Figure S8: Hazard ratios of ICU admission (COVID-19-specific) for COVID-19 cases infected with the Omicron versus Delta variants, as Figure 3F (adjusted for the required, highly desired and desired set of adjustment variables, using stratification for calendar week and regression adjustment for exact calendar date), by age group.

Note: Some countries observed no ICU admissions in some age groups, and were therefore not included in all age group analyses: this included Denmark for cases aged 0-19 and 40-59 years, Luxembourg for cases aged <40 years, Norway for cases aged 40-49 or 60-69 years, and Scotland for cases aged <40 years. The results for these countries and age groups were therefore not included in the sub-analyses.

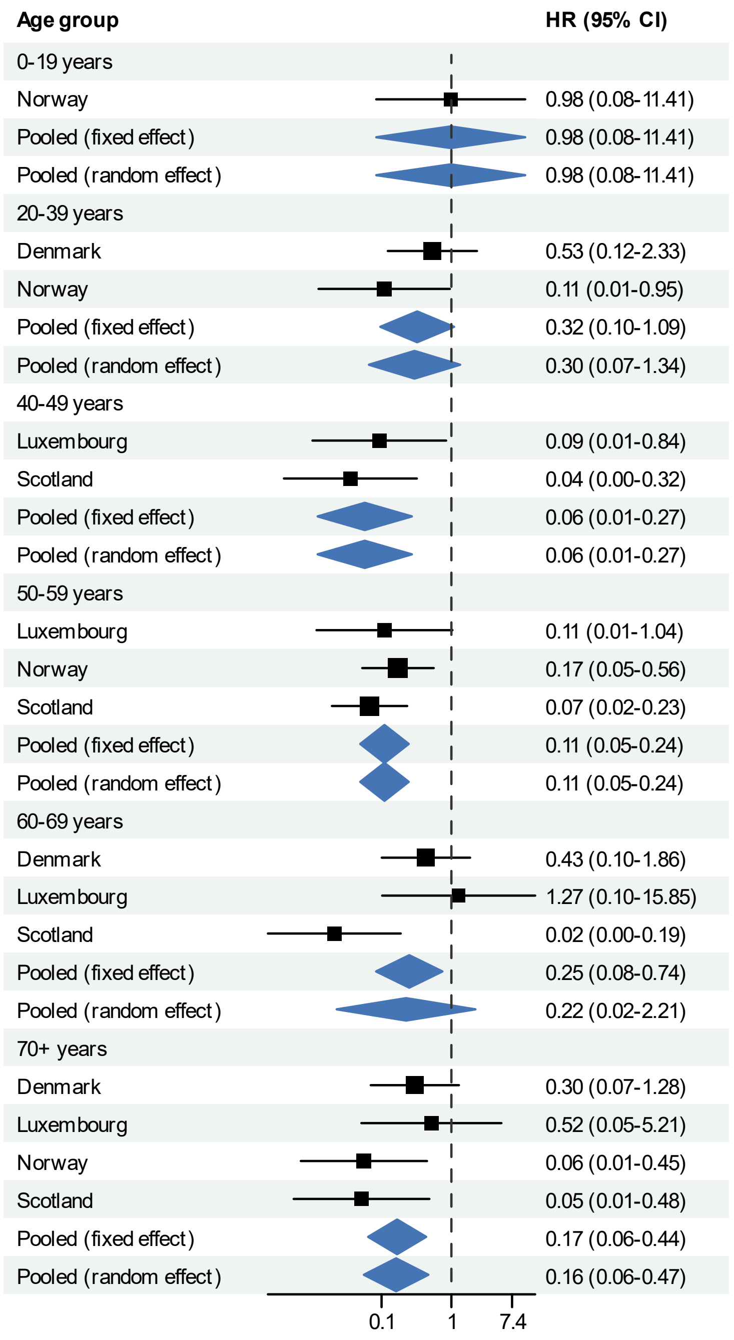
Age group
HR (95% CI)
0-19 years
Norway
0.98 (0.08-11.41)
Pooled (fixed effect)
0.98 (0.08-11.41)
Pooled (random effect)
0.98 (0.08-11.41)
20-39 years
Denmark
0.53 (0.12-2.33)
Norway
0.11 (0.01-0.95)
Pooled (fixed effect)
0.32 (0.10-1.09)
Pooled (random effect)
0.30 (0.07-1.34)
40-49 years
Luxembourg
0.09 (0.01-0.84)
Scotland
0.04 (0.00-0.32)
Pooled (fixed effect)
0.06 (0.01-0.27)
Pooled (random effect)
0.06 (0.01-0.27)
50-59 years
Luxembourg
0.11 (0.01-1.04)
Norway
0.17 (0.05-0.56)
Scotland
0.07 (0.02-0.23)
Pooled (fixed effect)
0.11 (0.05-0.24)
Pooled (random effect)
0.11 (0.05-0.24)
60-69 years
Denmark
0.43 (0.10-1.86)
Luxembourg
1.27 (0.10-15.85)
Scotland
0.02 (0.00-0.19)
Pooled (fixed effect)
0.25 (0.08-0.74)
Pooled (random effect)
0.22 (0.02-2.21)
70+ years
Denmark
0.30 (0.07-1.28)
Luxembourg
0.52 (0.05-5.21)
Norway
0.06 (0.01-0.45)
Scotland
0.05 (0.01-0.48)
Pooled (fixed effect)
0.17 (0.06-0.44)
Pooled (random effect)
0.16 (0.06-0.47)
0.1
1
7.4

Supplementary Figure S9: Hazard ratios of death (COVID-19-specific, where available, or otherwise due to any cause) for COVID-19 cases infected with the Omicron versus Delta variants, as Figure 4F (adjusted for the required, highly desired and desired set of adjustment variables, using stratification for calendar week and regression adjustment for exact calendar date), by age group.

Note: Some countries observed no deaths in some age groups, and were therefore not included in all age group analyses: this included Denmark for cases aged <60 years, Luxembourg for cases aged <60 years, Norway for cases aged <50 years, and Scotland for cases aged <40 years. The results for these countries and age groups were therefore not included in the sub-analyses.

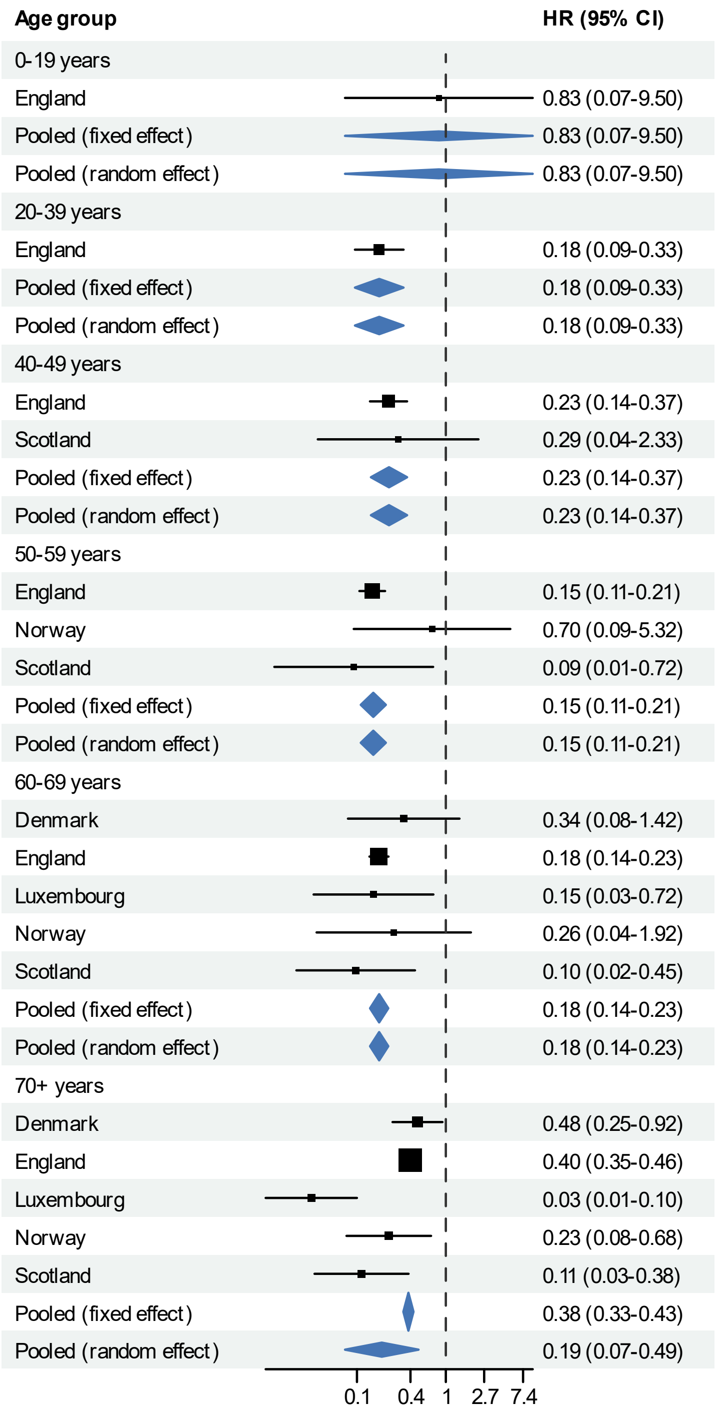

Age group
HR (95% CI)
0-19 years
England 0.83 (0.07-9.50)
Pooled (fixed effect) 0.83 (0.07-9.50)
Pooled (random effect) 0.83 (0.07-9.50)
20-39 years
England 0.18 (0.09-0.33)
Pooled (fixed effect) 0.18 (0.09-0.33)
Pooled (random effect) 0.18 (0.09-0.33)
40-49 years
England 0.23 (0.14-0.37)
Scotland 0.29 (0.04-2.33)
Pooled (fixed effect) 0.23 (0.14-0.37)
Pooled (random effect) 0.23 (0.14-0.37)
50-59 years
England 0.15 (0.11-0.21)
Norway 0.70 (0.09-5.32)
Scotland 0.09 (0.01-0.72)
Pooled (fixed effect) 0.15 (0.11-0.21)
Pooled (random effect) 0.15 (0.11-0.21)
60-69 years
Denmark 0.34 (0.08-1.42)
England 0.18 (0.14-0.23)
Luxembourg 0.15 (0.03-0.72)
Norway 0.26 (0.04-1.92)
Scotland 0.10 (0.02-0.45)
Pooled (fixed effect) 0.18 (0.14-0.23)
Pooled (random effect) 0.18 (0.14-0.23)
70+ years
Denmark 0.48 (0.25-0.92)
England 0.40 (0.35-0.46)
Luxembourg 0.03 (0.01-0.10)
Norway 0.23 (0.08-0.68)
Scotland 0.11 (0.03-0.38)
Pooled (fixed effect) 0.38 (0.33-0.43)
Pooled (random effect) 0.19 (0.07-0.49)
0.1
0.4
1
2.7
7.4

Supplementary Figure S10: Hazard ratios of ICU admission (COVID-19-specific) for COVID-19 cases infected with the Omicron versus Delta variants, as Figure 3F (adjusted for the required, highly desired and desired set of adjustment variables, using stratification for calendar week and regression adjustment for exact calendar date), by vaccination status.

Note: Some countries observed no ICU admissions in some vaccination subgroups, and were therefore not included in all vaccination status subgroup analyses.

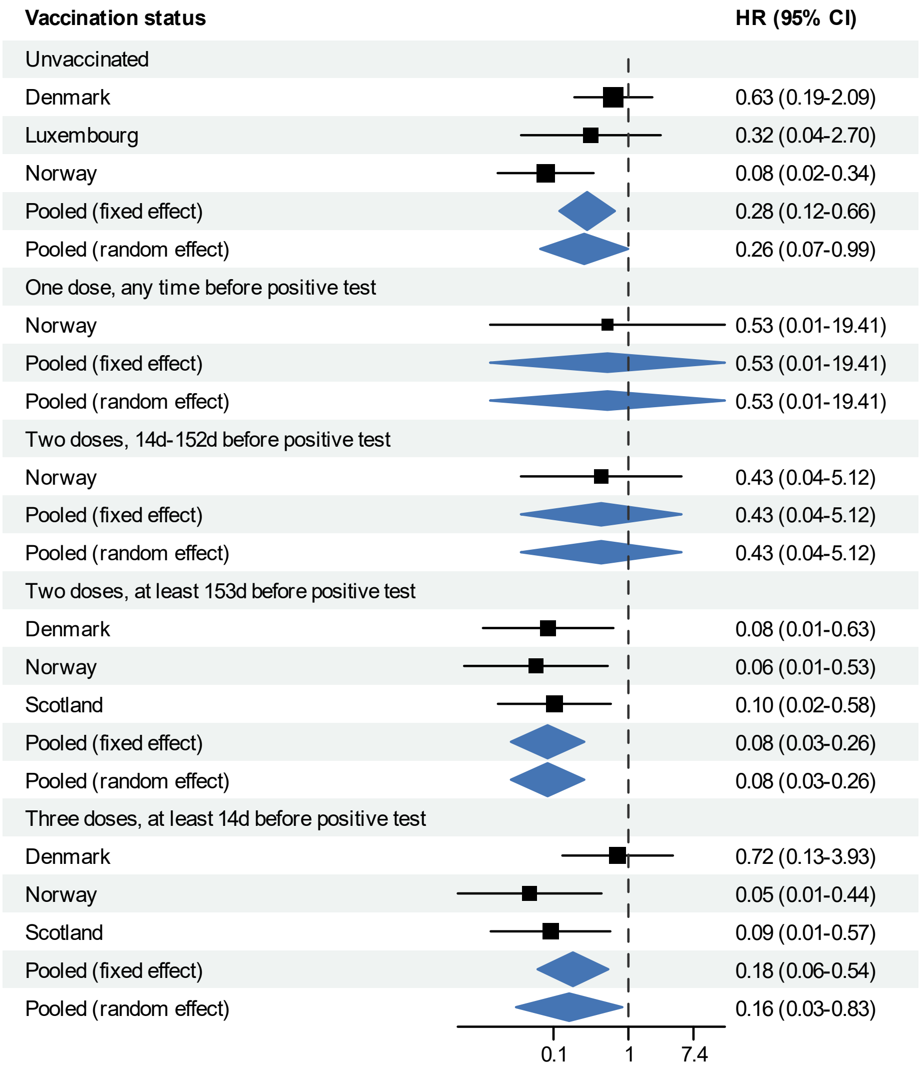

Supplementary Figure S11: Hazard ratios of death (COVID-19-specific, where available, or otherwise due to any cause) for COVID-19 cases infected with the Omicron versus Delta variants, as Figure 4F (adjusted for the required, highly desired and desired set of adjustment variables, using stratification for calendar week and regression adjustment for exact calendar date), by vaccination status.

Note: Some countries observed no deaths in some vaccination subgroups, and were therefore not included in all vaccination status subgroup analyses.

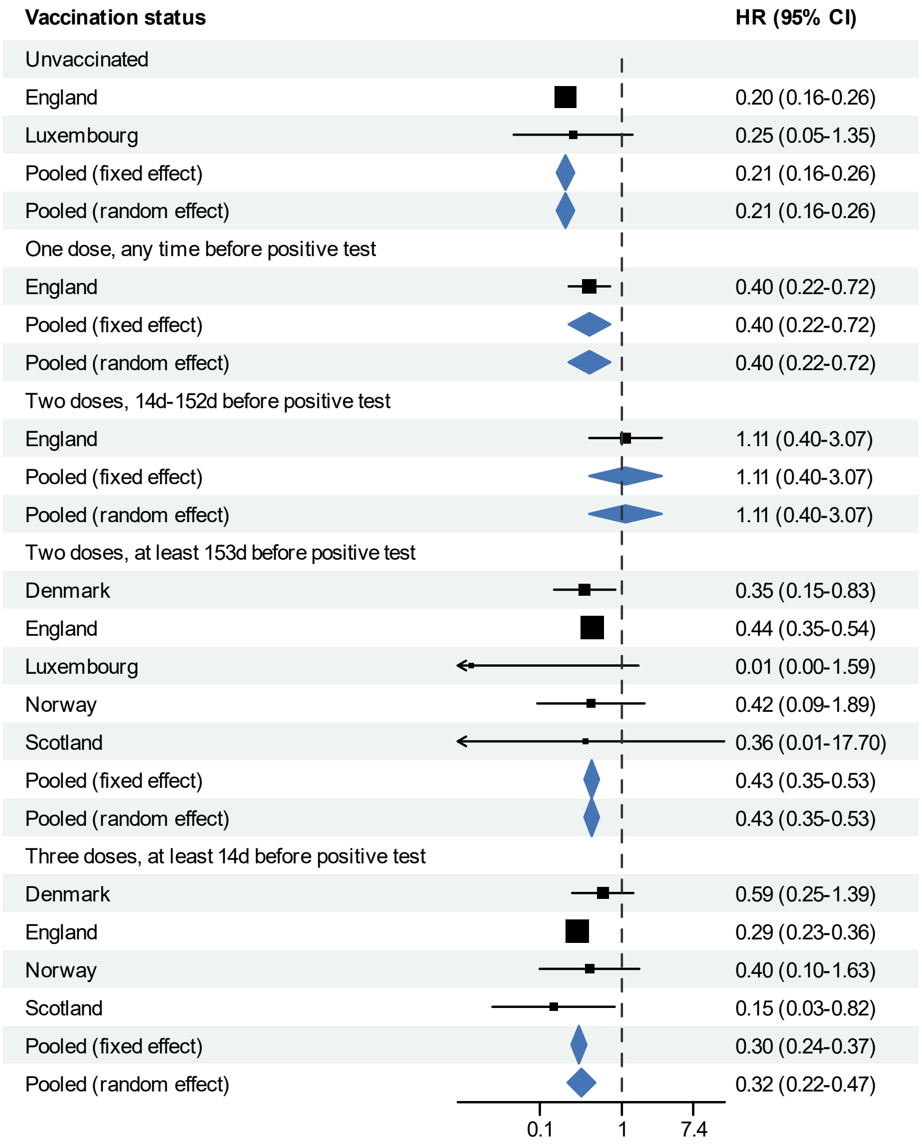

Supplementary Figure S12: Sensitivity analysis to assess the effect of epidemic phase bias [5]. Hazard ratios of hospital admission (COVID-19-specific, where available, or otherwise due to any cause) for COVID-19 cases infected with the Omicron versus Delta variants, as Figure 2F (adjusted for the required, highly desired and desired set of adjustment variables, using stratification for calendar week and regression adjustment for exact calendar date), after adjusting for the effect of epidemic phase bias.

Epidemic phase bias may occur when comparing two virus variants that are in different phases of incidence growth or decline. When the incidence of a variant is growing, cases who test positive with the variant tend to include a higher proportion of individuals with relatively shorter time since infection, and cases who test positive with a variant whose incidence is declining tend to include individuals with relatively longer time since infection. This might bias estimates of the relative risk between the variants when date of infection is unknown and adjustments are instead based on date of positive test, if infection severity if associated with shorter times from infection to test. To assess the potential impact of such bias, Seaman and colleagues [5] proposed a sensitivity analysis where a proxy adjustment date for date of infection is constructed based on assumed range for the mean difference in time from infection to positive test between those who do not experience severe disease and those who do.

The plot shows the results assuming a range of the mean difference of between 0 days and 4 days.

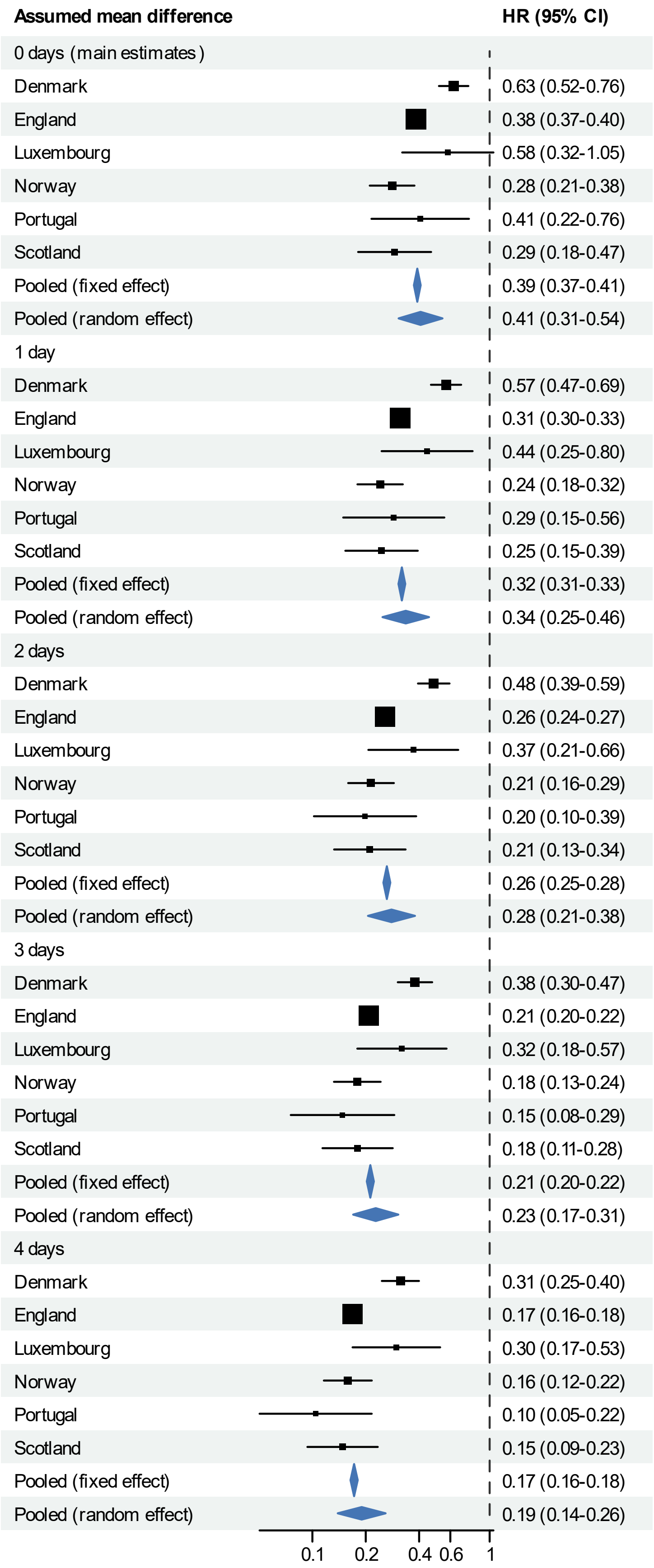
Assumed mean difference
HR (95% CI)
0 days (main estimates)
Denmark 0.63 (0.52-0.76)
England 0.38 (0.37-0.40)
Luxembourg 0.58 (0.32-1.05)
Norway 0.28 (0.21-0.38)
Portugal 0.41 (0.22-0.76)
Scotland 0.29 (0.18-0.47)
Pooled (fixed effect) 0.39 (0.37-0.41)
Pooled (random effect) 0.41 (0.31-0.54)
1 day
Denmark 0.57 (0.47-0.69)
England 0.31 (0.30-0.33)
Luxembourg 0.44 (0.25-0.80)
Norway 0.24 (0.18-0.32)
Portugal 0.29 (0.15-0.56)
Scotland 0.25 (0.15-0.39)
Pooled (fixed effect) 0.32 (0.31-0.33)
Pooled (random effect) 0.34 (0.25-0.46)
2 days
Denmark 0.48 (0.39-0.59)
England 0.26 (0.24-0.27)
Luxembourg 0.37 (0.21-0.66)
Norway 0.21 (0.16-0.29)
Portugal 0.20 (0.10-0.39)
Scotland 0.21 (0.13-0.34)
Pooled (fixed effect) 0.26 (0.25-0.28)
Pooled (random effect) 0.28 (0.21-0.38)
3 days
Denmark 0.38 (0.30-0.47)
England 0.21 (0.20-0.22)
Luxembourg 0.32 (0.18-0.57)
Norway 0.18 (0.13-0.24)
Portugal 0.15 (0.08-0.29)
Scotland 0.18 (0.11-0.28)
Pooled (fixed effect) 0.21 (0.20-0.22)
Pooled (random effect) 0.23 (0.17-0.31)
4 days
Denmark 0.31 (0.25-0.40)
England 0.17 (0.16-0.18)
Luxembourg 0.30 (0.17-0.53)
Norway 0.16 (0.12-0.22)
Portugal 0.10 (0.05-0.22)
Scotland 0.15 (0.09-0.23)
Pooled (fixed effect) 0.17 (0.16-0.18)
Pooled (random effect) 0.19 (0.14-0.26)
0.1
0.2
0.4
0.6
1

Figure S13: Hazard ratios of hospital admission (COVID-19-specific, where available, or otherwise due to any cause) for COVID-19 cases infected with the Omicron versus Delta variants, by country and pooled across countries using a fixed effect and a random effect model; as Figure 2, but restricted to protocol-compliant cases with reported Delta variant information in Denmark (see Methods).

(A). Adjusted for the required set of adjustment variables, using stratification for exact calendar date.
(B). Adjusted for the required and highly desired set of adjustment variables, using stratification for exact calendar date.
(C). Adjusted for the required, highly desired and desired set of adjustment variables, using stratification for exact calendar date.
(D). As (A), but using stratification for calendar week and regression adjustment for exact calendar date.
(E). As (B), but using stratification for calendar week and regression adjustment for exact calendar date.
(F). As (C), but using stratification for calendar week and regression adjustment for exact calendar date.

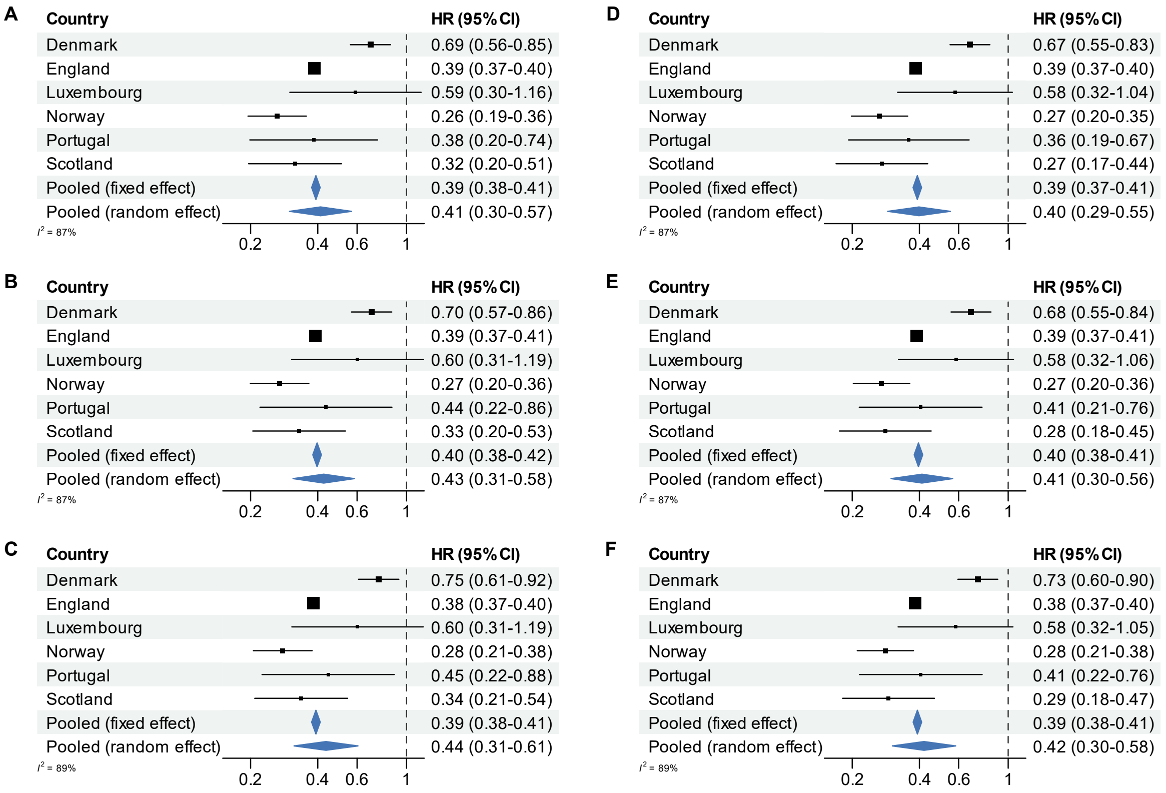

References, Supplementary material

1. Schønning K, Dessau RB, Jensen TG, Thorsen NM, Wiuff C, Nielsen L, et al. Electronic reporting of diagnostic laboratory test results from all healthcare sectors is a cornerstone of national preparedness and control of COVID-19 in Denmark. APMIS. 2021;129(7):438-51.
2. Spiess K. Rapid and flexible RT-qPCR surveillance platforms to detect SARS-CoV-2 mutations. Microbiology Spectrum. 2022 (in press).
3. George F. Primeiro apontamento sobre o Sistema de Informacão dos Certificados de Óbito (SICO). Revista Portuguesa de Saúde Pública. 2014;32(1):1-2.
4. Veneti L, Boas H, Brathen Kristoffersen A, Stalcrantz J, Bragstad K, Hungnes O, et al. Reduced risk of hospitalisation among reported COVID-19 cases infected with the SARS-CoV-2 Omicron BA.1 variant compared with the Delta variant, Norway, December 2021 to January 2022. Euro Surveill. 2022;27(4).
5. Seaman SR, Nyberg T, Overton CE, Pascall DJ, Presanis AM, De Angelis D. Adjusting for time of infection or positive test when estimating the risk of a post-infection outcome in an epidemic. Statistical Methods in Medical Research. 2022;31(10):1942-58.
