# Supplementary material for: A standardised protocol for relative SARS-CoV-2 variant severity assessment, applied to Omicron BA.1 and Delta in six European countries, October 2021 to February 2022
Source: Euro Surveill. 2023 Sep 7;28(36):2300048. doi: 10.2807/1560-7917.ES.2023.28.36.2300048 (PMC10486193; doi:10.2807/1560-7917.ES.2023.28.36.2300048)
Supplement: Supplementary Material 3 [file 23-00048_NYBERG_Supplement3.pdf]

# Supplement 3

## Power estimation

This supplementary material is hosted by *Eurosurveillance* as supporting information alongside the article “A standardised protocol for relative SARS-CoV-2 variant severity assessment, applied to Omicron BA.1 and Delta in six European countries, October 2021 to February 2022”, on behalf of the authors, who remain responsible for the accuracy and appropriateness of the content. The same standards for ethics, copyright, attributions and permissions as for the article apply. Supplements are not edited by *Eurosurveillance* and the journal is not responsible for the maintenance of any links or email addresses provided therein.

## Methods

Statistical power in the context of relative risk estimation refer to the probability to detect a statistically significant difference in risk between the comparison groups when a true difference exists. Using previously proposed approximations for calculation of the power for meta-analyses based on random effects models [1], and the arcsine approximation of the standardised effect size for comparison of proportions between two groups [2], we here provide a range of expected statistical power values based on example scenarios. These calculations are simplifications that assume equal number of cases with each of two compared virus variants, equal number of cases per included country, and no confounding factors that require adjustments. The scenarios assume: a true expected relative risk (RR) of either 1.5 or 1.3 (risk of the outcome of 0.75% or 0.65%, respectively, for cases with a new variant compared to 0.50% for cases with a reference variant); that either 5 or 10 countries are included; that the number of cases with each variant is either 2,000, 5,000 or 10,000 per country; and that the heterogeneity between countries as measured by $I^2$ is either 0%, 20%, 50% or 80%. For comparison, the corresponding power is shown based on a scenario that all data are instead pooled into a single dataset and the estimation is instead based on standard non-meta-analysis methods. All calculations assume two-sided tests at 5% significance level.

## Results

Supplementary Table S4 shows the results of the power calculations. Under scenarios that assumed a RR=1.5 and with no to moderate heterogeneity between countries ($I^2$≤50%), power of 80% or higher was expected in scenarios with at least 5,000 cases with each variant per country when 5 countries were assumed, or at least 2,000 cases with each variant per country when 10 countries were assumed. For the corresponding scenarios that assumed high heterogeneity ($I^2$=80%), power of 80% or higher was expected with 10,000 cases with each variant per country when 5 countries were assumed, or at least 5,000 cases with each variant per country when 10 countries were assumed. Under the alternative scenarios that assumed a RR=1.3 and with no to moderate heterogeneity between countries ($I^2$≤50%), power of 80% or higher was expected in scenarios with at least 10,000 cases with each variant per country when 5 countries were assumed, or at least 5,000 cases with each variant per country when 10 countries were assumed. For the corresponding scenarios that assumed high heterogeneity ($I^2$=80%), power ≥80% was only expected with 10,000 cases with each variant per country when 10 countries were assumed.

As expected, under all scenarios where there was no heterogeneity of the estimates between countries, the pooled meta-analysis estimates had equal power to the corresponding power from an analysis where all data were instead pooled into a single dataset.

**Supplementary Table S4.** Expected statistical power when random effects meta-analysis is used to pool the separate relative risks (RRs) from several countries of an outcome between cases with two different virus variants. The table shows the power under example scenarios by: assumed relative risk (RR); number of included countries; number of cases with each variant per country; and, heterogeneity between the countries' RR estimates.

| Scenario | Number of countries | N with each variant per country | Power | | | | |
|---|---|---|---|---|---|---|---|
| | | | **All data pooled to a single dataset, no heterogeneity ($I^2$=0%)** | **Meta-analysis of estimates from each country, no heterogeneity ($I^2$=0%)** | **Meta-analysis of estimates from each country, low heterogeneity ($I^2$=20%)** | **Meta-analysis of estimates from each country, moderate heterogeneity ($I^2$=50%)** | **Meta-analysis of estimates from each country, high heterogeneity ($I^2$=80%)** |
| True RR=1.5[a] | 5 countries | 2,000 | 61.6% | 61.6% | 59.4% | 53.9% | 39.0% |
| | 5 countries | 5,000 | 94.6% | 94.6% | 93.6% | 90.2% | 75.7% |
| | 5 countries | 10,000 | 99.9% | 99.9% | 99.8% | 99.6% | 96.4% |
| | 10 countries | 2,000 | 89.0% | 89.0% | 88.3% | 85.6% | 76.9% |
| | 10 countries | 5,000 | 99.9% | 99.9% | 99.9% | 99.8% | 98.9% |
| | 10 countries | 10,000 | >99.9% | >99.9% | >99.9% | >99.9% | >99.9% |
| True RR=1.3[b] | 5 countries | 2,000 | 29.0% | 29.0% | 27.8% | 24.9% | 18.1% |
| | 5 countries | 5,000 | 60.4% | 60.4% | 58.3% | 52.8% | 38.1% |
| | 5 countries | 10,000 | 88.2% | 88.2% | 86.6% | 81.8% | 64.9% |
| | 10 countries | 2,000 | 51.1% | 51.1% | 50.1% | 47.4% | 39.0% |
| | 10 countries | 5,000 | 88.2% | 88.2% | 87.4% | 85.0% | 75.7% |
| | 10 countries | 10,000 | 99.4% | 99.4% | 99.2% | 98.9% | 96.4% |

[a] The risk of the outcome is assumed to be 0.75% for cases with the higher risk variant compared to 0.50% for cases with the reference variant.

[b] The risk of the outcome is assumed to be 0.65% for cases with the higher risk variant compared to 0.50% for cases with the reference variant.

# References, Supplement C

1. Valentine JC, Pigott TD, Rothstein HR. How Many Studies Do You Need?:A Primer on Statistical Power for Meta-Analysis. Journal of Educational and Behavioral Statistics. 2010;35(2):215-47.
2. Champely S. pwr: Basic Functions for Power Analysis. R package version 1.3-0. 2020.
